# A novel method for the determination of field output factors and output correction factors for small static fields for six diodes and a microdiamond detector in megavoltage photon beams

**DOI:** 10.1002/mp.13318

**Published:** 2018-12-24

**Authors:** Božidar Casar, Eduard Gershkevitsh, Ignasi Mendez, Slaven Jurković, M. Saiful Huq

**Affiliations:** ^1^ Department for Dosimetry and Quality of Radiological Procedures Institute of Oncology Ljubljana Zaloška 2 1000 Ljubljana Slovenia; ^2^ Medical Physics Service North Estonia Medical Centre J. Sütiste tee 19 13419 Tallinn Estonia; ^3^ Medical Physics Department University Hospital Rijeka Krešimirova 42 51000 Rijeka Croatia; ^4^ Department of Radiation Oncology University of Pittsburgh School of Medicine and UPMC Hillman Cancer Center Pittsburgh PA USA

**Keywords:** W1 scintillator, EBT3 films, diodes, microDiamond, small fields

## Abstract

**Purpose:**

The goal of this work is to provide a large and consistent set of data for detector‐specific output correction factors, $k_{Q_{\mathrm{clin}},Q_{\mathrm{ref}}}^{\;f_{\mathrm{clin}},\;f_{\mathrm{ref}}}$, for small static fields for seven solid‐state detectors and to determine field output factors, $\Omega_{Q_{\mathrm{clin}},Q_{\mathrm{ref}}}^{\;f_{\mathrm{clin}},\;f_{\mathrm{ref}}}$, using EBT3 radiochromic films and W1 plastic scintillator as reference detectors on two different linear accelerators and four megavoltage photon beams. Consistent measurement conditions and recommendations given in the International Code of Practice TRS‐483 for small‐field dosimetry were followed throughout the study.

**Methods:**

$\Omega_{Q_{\mathrm{clin}},Q_{\mathrm{ref}}}^{\;f_{\mathrm{clin}},\;f_{\mathrm{ref}}}$ were determined on two linacs, Elekta Versa HD and Varian TrueBeam, for 6 and 10 MV beams with and without flattening filter and for nine fields ranging from 0.5 × 0.5 cm^2^ to 10 × 10 cm^2^. Signal readings obtained with EBT3 radiochromic films and W1 plastic scintillator were fitted by an analytical function. Volume averaging correction factors, determined from two‐dimensional (2D) dose matrices obtained with EBT3 films and fitted to bivariate Gaussian function, were used to correct measured signals. $k_{Q_{\mathrm{clin}},Q_{\mathrm{ref}}}^{\;f_{\mathrm{clin}},\;f_{\mathrm{ref}}}$ were determined empirically for six diodes, IBA SFD, IBA Razor, PTW 60008 P, PTW 60012 E, PTW 60018 SRS, and SN EDGE, and a PTW 60019 microDiamond detector.

**Results:**

Field output factors and detector‐specific $k_{Q_{\mathrm{clin}},Q_{\mathrm{ref}}}^{\;f_{\mathrm{clin}},\;f_{\mathrm{ref}}}$ are presented in the form of analytical functions as well as in the form of discrete values. It is found that in general, for a given linac, small‐field output factors need to be determined for every combination of beam energy and filtration (WFF or FFF) and field size as the differences between them can be statistically significant (*P* < 0.05). For different beam energies, the present data for $k_{Q_{\mathrm{clin}},Q_{\mathrm{ref}}}^{\;f_{\mathrm{clin}},\;f_{\mathrm{ref}}}$ are found to differ significantly (*P* < 0.05) from the corresponding data published in TRS‐483 mostly for the smallest fields (<1.5 cm). For the PTW microDiamond detector, statistically significant differences (*P* < 0.05) between $k_{Q_{\mathrm{clin}},Q_{\mathrm{ref}}}^{\;f_{\mathrm{clin}},\;f_{\mathrm{ref}}}$ values were found for all investigated beams on an Elekta Versa HD linac for field sizes 0.5 × 0.5 cm^2^ and 0.8 × 0.8 cm^2^. Significant differences in $k_{Q_{\mathrm{clin}},Q_{\mathrm{ref}}}^{\;f_{\mathrm{clin}},\;f_{\mathrm{ref}}}$ between beams of a given energy but with and without flattening filters are found for measurements made in small fields (<1.5 cm) at a given linac. Differences in $k_{Q_{\mathrm{clin}},Q_{\mathrm{ref}}}^{\;f_{\mathrm{clin}},\;f_{\mathrm{ref}}}$ are also found when measurements are made at different linacs using the same beam energy filtration combination; for the PTW microDiamond detector, these differences were found to be around 6% and were considered as significant.

**Conclusions:**

Selection of two reference detectors, EBT3 films and W1 plastic scintillator, and use of an analytical function, is a novel approach for the determination of $\Omega_{Q_{\mathrm{clin}},Q_{\mathrm{ref}}}^{\;f_{\mathrm{clin}},\;f_{\mathrm{ref}}}$ for small static fields in megavoltage photon beams. Large set of $k_{Q_{\mathrm{clin}},Q_{\mathrm{ref}}}^{\;f_{\mathrm{clin}},\;f_{\mathrm{ref}}}$ data for seven solid‐state detectors and four beam energies determined on two linacs by a single group of researchers can be considered a valuable supplement to the literature and the TRS‐483 dataset.

## 1 Introduction

In recent years, the availability of modern technologies has facilitated the use of radiotherapy techniques such as intensity modulated radiation therapy (IMRT), volumetric modulated arc therapy (VMAT), stereotactic radiosurgery (SRS), and stereotactic body radiation therapy (SBRT) for the treatment of cancer patients using external beam radiation therapy. These techniques use many small fields for the planning and delivery of prescription dose. There are three physical conditions, which determine if a megavoltage (MV) photon beam can be considered as small: a loss or lack of lateral charged‐particle equilibrium (LCPE), partial occlusion of the primary radiation source by the machine collimating devices, and a mismatch between the size of the detector and the field dimensions. Only one of these three conditions needs to be fulfilled to designate a particular photon field as small. Until the recent joint publication of the International Code of Practice (CoP) TRS‐483 for reference and relative dosimetry by the International Atomic Energy Agency (IAEA) and the American Association of Physicists in Medicine (AAPM),^1^ no national or international guidance for performing reference and relative dosimetry in small fields was available to the practicing medical physicists. This resulted in the occurrence of dosimetric errors in many clinics and many accidents involving the incorrect use of small fields were reported in the literature.^2^, ^3^ In this respect, it should be noted that the IAEA TRS‐398 or AAPM TG‐51 and TG‐51 Addendum protocols provided guidance for reference dosimetry in conventional reference fields and do not provide guidance for dosimetry in small fields.^4^, ^5^, ^6^

The IAEA and AAPM jointly published TRS‐483 Code of Practice^1^ which provided an extensive set of data for detector‐specific output correction factors for the determination of field output factors in small fields. These data were based on both experimental and Monte Carlo (MC) calculated data that were available in the literature at the time of writing the CoP.

For the determination of field output factors, the TRS‐483 followed the formalism proposed by Alfonso et al.^7^ For a particular clinical field $f_{\mathrm{clin}}$ and reference field $f_{\mathrm{ref}}$ (10 cm × 10 cm), the field output factor $\Omega_{Q_{\mathrm{clin}},Q_{\mathrm{ref}}}^{f_{\mathrm{clin}},f_{\mathrm{ref}}}$ is determined from the ratio of doses in both fields, given by

(1) $$\Omega_{Q_{\mathrm{clin}},Q_{\mathrm{ref}}}^{f_{\mathrm{clin}},f_{\mathrm{ref}}}=\frac{D_{w,Q_{\mathrm{clin}}}^{f_{\mathrm{clin}}}}{D_{w,Q_{\mathrm{ref}}}^{f_{\mathrm{ref}}}}$$

where $Q_{\mathrm{clin}}$ and $Q_{\mathrm{ref}}$ denote the beam quality in the clinical and reference fields, respectively. Note that both Alfonso et al. and TRS‐483 use the notation msr (machine specific reference) to denote machine‐specific reference fields in machines that cannot set the conventional reference field 10 cm × 10 cm.

For large fields, field output factors can be approximated by the ratio between detector readings $M_{Q_{\mathrm{clin}}}^{f_{\mathrm{clin}}}$ and $M_{Q_{\mathrm{ref}}}^{f_{\mathrm{ref}}}$ in clinical and reference fields, respectively. However, this approximation does not hold true in the case of small fields, where an output correction factor $k_{Q_{\mathrm{clin}},Q_{\mathrm{ref}}}^{f_{\mathrm{clin}},f_{\mathrm{ref}}}$ is necessary to take into account the differences in the response of a detector in the clinical and reference fields.^1^ Thus, the field output factor is supplied by Eq. (2):

(2) $$\Omega_{Q_{\mathrm{clin}},Q_{\mathrm{ref}}}^{f_{\mathrm{clin}},f_{\mathrm{ref}}}=\frac{M_{Q_{\mathrm{clin}}}^{f_{\mathrm{clin}}}}{M_{Q_{\mathrm{ref}}}^{f_{\mathrm{ref}}}}k_{Q_{\mathrm{clin}},Q_{\mathrm{ref}}}^{f_{\mathrm{clin}},f_{\mathrm{ref}}}$$

Detector‐specific output correction factors $k_{Q_{\mathrm{clin}},Q_{\mathrm{ref}}}^{f_{\mathrm{clin}},f_{\mathrm{ref}}}$ depend on many factors such as the perturbation of particle fluence and volume averaging effects, in particular, on the field size, detector type, and the design, size, and non‐water equivalency of most of the detectors. It is also of interest to know whether $k_{Q_{\mathrm{clin}},Q_{\mathrm{ref}}}^{f_{\mathrm{clin}},f_{\mathrm{ref}}}$ depends on the way the field is collimated on the linear accelerator (e.g., using MLC, jaws or combination of both) and on the type of accelerator.

To derive output correction factors for small fields from the data published in the literature, TRS‐483 considered three types of datasets. One of these datasets considered the reference detectors to be perturbation free except for volume averaging. The main characteristics of these detectors are their near water equivalency, with radiological properties close to the corresponding values for water, which also have weak or negligible energy dependence in the MV radiotherapy photon beams. Examples are some passive detectors such as alanine and radiochromic films. The only commercially available active dosimeter with properties close to that of water and weak energy dependence is the plastic scintillator detector.

Response of detectors in small fields and the determination of detector‐specific output correction factors have been extensively investigated for a range of detectors by several research groups, using one of the following three techniques: (a) empirical approach, where uncorrected signal ratios were determined and compared to the field output factors determined with reference detectors,^8^, ^9^, ^10^, ^11^, ^12^, ^13^, ^14^, ^15^, ^16^, ^17^, ^18^, ^19^, ^20^, ^21^, ^22^, ^23^, ^24^, ^25^, ^26^ (b) numerical approach, where $k_{Q_{\mathrm{clin}},Q_{\mathrm{ref}}}^{f_{\mathrm{clin}},f_{\mathrm{ref}}}$ were determined with MC simulations,^27^, ^28^, ^29^, ^30^, ^31^, ^32^ and (c) semi‐empirical approach which combines both, measurements and numerical/analytical calculations, and where $k_{Q_{\mathrm{clin}},Q_{\mathrm{ref}}}^{f_{\mathrm{clin}},f_{\mathrm{ref}}}$ were the most commonly determined through the comparison of measured uncorrected detector's signal ratios with MC calculated field output factors.^25^, ^33^, ^34^, ^35^, ^36^ There are advantages and disadvantages of each of these approaches.^37^ However, since the numerical MC calculations have to be verified and validated with measurements, in this paper, an empirical approach for the determination of $\Omega_{Q_{\mathrm{clin}},Q_{\mathrm{ref}}}^{f_{\mathrm{clin}},f_{\mathrm{ref}}}$ using two reference detectors was used. Thus, the $k_{Q_{\mathrm{clin}},Q_{\mathrm{ref}}}^{f_{\mathrm{clin}},f_{\mathrm{ref}}}$ were determined using an empirical approach.

Experimental determination of field output factors in small fields is challenging because detectors that can be used to accurately determine field output factors, without requiring corrections for non‐water equivalency of their sensitive measuring volume and volume averaging due to their finite size, are presently not commercially available. Furthermore, for very small fields with dimensions below about 2 cm, small positional uncertainties can lead to significant uncertainties in the measurement results. This means that there are no ideal detectors for measurements of field output factors in small fields. Although a large amount of experimental and numerical data for field output factors and output correction factors for different detectors are available in the literature, there is considerable scatter of such data for the smallest field sizes; additionally, lack of homogeneity in the measurement setup and the definition of field size make interpretation of such data very challenging.^1^

One of the goals of the present study was to provide a consistent set of data for detector‐specific output correction factors and field output factors determined using consistent measurement conditions and recommendations given in TRS‐483. Analytic functions for field output factors have been derived from measured data of field output factors on two different linear accelerators for 6 and 10 MV photon beams with flattening filter (WFF) and flattening filter‐free beams (FFF) using radiochromic films and plastic scintillators as reference detectors. These two detectors are referred to as perturbation free,^1^ except for volume averaging which was appropriately considered.

A second goal was to provide a large set of consistent detector‐specific output correction factors for seven solid‐state detectors, six commonly used diodes and a synthetic microdiamond, for investigated MV small beams on two linear accelerators (hereafter abbreviated as linacs), both in the form of an analytical function as well as in the form of discrete values based on empirical data. The present datasets and results will provide a valuable supplement to the dataset given in TRS‐483 and serve to validate the dataset given in TRS‐483.

A third goal was to verify whether output correction factors determined from measurements made on different c‐arm linacs made by different vendors using different solid‐state detectors for different combinations of field size, beam energy and filtration (WFF or FFF) are the same.

Throughout this study, the same experimental setup was used for all measurements for all detectors, following the recommendations given in TRS‐483. Field output correction factors and detector‐specific output correction factors, presented in analytical form in this study, may serve as a reference dataset for comparison with other studies, and for small fields not used explicitly in our measurements, considering similar or comparable experimental setup and conditions.

## 2 Methods and materials

### 2.A. Experimental setup

Dosimetry measurements were performed at two hospitals on two different linacs, Elekta Versa HD™ (Elekta AB, Stockholm, Sweden) and Varian TrueBeam™ (Varian Medical Systems, Palo Alto, CA, USA), using high‐energy photon beams of energies 6 and 10 MV. Beams with flattening filters WFF as well as FFF beams, denoted hereafter as 6 MV WFF, 6 MV FFF, 10 MV WFF, and 10 MV FFF, were used for all measurements. The measurement geometry consisted of an isocentric setup with a source‐to‐surface (SSD) distance of 90 cm and a depth of 10 cm and gantry at 0°. For each point measurement, 100 MU were delivered, for nine square fields with nominal side lengths of 0.5, 0.8, 1.0, 1.5, 2.0, 3.0, 4.0, 5.0, and 10.0 cm. The 10 × 10 cm^2^ field size was used as the reference field size for the calculation of field output factors. At least three measurements were taken for each specific setup unless otherwise specified. For all point measurements in water, a reference class PTW Unidos^*webline*^ (PTW, Freiburg, Germany) electrometer was used throughout the study.

In this study, the dose–response of nine types of detectors was investigated. These consisted of a plastic scintillator Exradin W1 (Standard Imaging, Middleton, WI, USA), radiochromic film EBT3 (Ashland Inc., Wayne, NJ, USA), and seven solid‐state detectors, that is, six diodes and a synthetic microdiamond detector. For the solid‐state detectors, the same detectors with the same serial numbers were used for measurements in both linacs; EBT3 films from two different lots were used for measurements in the two linacs. The Exradin W1 plastic scintillator detector (W1 PSD) and EBT3 film detector were considered as the reference detectors and were used for the determination of an analytical function for field output factors. Based on this analytical function, detector‐specific output correction factors for the seven solid‐state detectors were obtained in the form of an analytical function and as discrete values.

A 3D water phantom (Blue Phantom 2, IBA Dosimetry, Schwarzenbruck, Germany) was used for the measurements in the first center on an Elekta Versa HD linac for all detectors except for EBT3 films, for which RW3 Solid Water phantom (PTW Freiburg, Freiburg, Germany) in the form of 30 × 30 cm^2^ slabs were used. Radiation fields were shaped with MLC in cross‐line (x) direction and with jaws in the in‐line (y) direction.

For all detectors, except for EBT3 films, a MP3‐M water phantom (PTW) was used for measurements in the second center equipped with a Varian TrueBeam linac. For EBT3 films, 30 × 30 cm^2^ slabs of Virtual Water (Standard Imaging) were used. To match the nominal field sizes with those on the Elekta Versa HD, radiation fields on the Varian TrueBeam were collimated using the linac jaws in both axes, x and y.

### 2.B. Radiochromic films EBT3

Weak energy dependence, near water‐equivalence, and high spatial resolution are the most important properties of radiochromic films that justify their use as a reference detector for relative dosimetry in small fields in MV photon beams. However, since uncertainties in data obtained using films can become significant from mishandling of films, careful handling of films is crucial to obtaining meaningful and accurate results. A strict protocol for film dosimetry was therefore followed throughout this study, from film cutting to final scanning.

#### 2.B.1. Film preparation and irradiation

Gafchromic EBT3 films from lot 04071601 were used for measurements on the Elekta Versa HD linac and from lot 06291702 on the Varian TrueBeam linac. From each lot, two films were employed for the purpose of calibration. Each calibration film was cut into seven strips with dimensions 20.32 × 3.5 cm^2^. One strip was left unexposed; the other six were irradiated with a 6 MV WFF beam. Calibration strips cut from both films were irradiated with 50, 100, 150, 200, 250, 300, 350, 400, 450, 500 (two pieces), and 600 MU on the Elekta Versa HD, and with 60, 120, 180, 240, 300, 360, 420, 480, 540, 600 (two pieces), and 720 MU on the Varian TrueBeam. Field sizes of 25 × 25 cm^2^ were used in order to expose them with homogeneous doses.

Field output factors were measured with three pieces of films for each combination of field size and photon energy. In total, 108 pieces of films (i.e., four photon energies times nine fields times three measurements) were irradiated on each linac. To reduce film uncertainties,^38^ central doses were kept to about 2 Gy or larger than that for all fields. On the Elekta Versa HD, films were irradiated with 500 MU for field output factor measurements, while on the Varian TrueBeam linac they were irradiated with 600 MU. The difference in MU reflects the fact that the Elekta linac was calibrated isocentrically (1 cGy/MU at SSD = 90 cm, depth 10 cm), while the Varian linac was calibrated at the depth of maximum ionization (1 cGy/MU at SSD = 100 cm, depth d_max_). Five unexposed films were also scanned to apply lateral corrections. The orientation of all films (i.e., calibration strips, films employed for field output factor measurements, and unexposed films) were marked to ensure consistency in scanning.

#### 2.B.2. Scanning

To reduce uncertainties, all films were scanned prior to and following irradiation. An Epson Expression 10000XL (Seiko Epson Corporation, Nagano, Japan) flatbed scanner was used for measurements made on the Elekta linac, while an Epson Expression 11000XL was used for measurements made on the Varian linac. Scanners were warmed up for at least 30 min before readings. A frame, cut out from a transparency sheet was employed to place films in a reproducible and cantered position on the scanner. Whenever there was a gap between the frame and the film pieces along the axis parallel to the lamp, it was closed with idle film pieces in order to minimize the cross‐talk effect.^39^ Before acquisitions and after long pauses, five empty scans were taken to stabilize the lamp. Each reading was repeated five times and the first scan was discarded. Scans were made in reflection mode and portrait orientation. Images were acquired with Epson Scan v3.49a software in 48‐bit RGB mode (16 bit per channel) with processing tools turned off, and saved as TIFF files. Resulting images were obtained as the average of repeated scans. In order to correct for inter‐scan variations, every film was scanned together with an unexposed calibration strip. Calibration films and field output factor film pieces were scanned with 50 and 150 dpi resolution, respectively. Lateral corrections were derived from the unexposed films and the calibration strips.^40^

#### 2.B.3. Dose calculation and field dimensions

Doses were computed using the Multigaussian model^40^ for radiochromic film dosimetry implemented in Radiochromic.com v3.0,^41^ after applying lateral and inter‐scan corrections. Data analysis was carried out with the R statistical computing environment.^42^

For each film, field dimensions along x and y directions were determined from measurements of the full width at half maximum (FWHM), and central doses as the mean of the dose values in a circular region of interest (ROI) drawn in the central part of the irradiated field with diameter of 0.5 mm. The center of the irradiated field was defined as the center of the dose profiles in both directions. Since central doses and field dimensions were calculated from measurements made on three different film pieces for each field, the final results were taken as the average of these three measurements.

### 2.C. Equivalent square small field size $S_{\mathrm{clin}}$

Nominal field sizes were converted to the equivalent square small field sizes $S_{\mathrm{clin}}$ for each field following the approach originally suggested by Cranmer‐Sargison et al.,^43^ used by other authors^16^ and adopted by TRS‐483 according to

(3) $$S_{\mathrm{clin}}=\sqrt{A\cdot B}$$

where A corresponds to the radiation field width (FWHM) in in‐line direction y and B (FWHM) for cross‐line direction x perpendicular to the former. $S_{\mathrm{clin}}$ has the same meaning as $FS_{\mathrm{eff}}$ in the original work by Cranmer‐Sargison et al. Radiation field widths A and B were determined from EBT3 film measurements as described earlier, and have been applied for all detectors used in the study. In this study, A and B correspond to the field widths defined by the FWHM at the measurement depth of 10 cm.

### 2.D. Exradin W1 plastic scintillator

The W1 PSD has radiological properties similar to EBT3 films with densities close to the values of water and belongs to the group of reference detectors, which are perturbation free except for volume averaging.^1^ The physical density of plastic scintillating fiber is 1.05 g/cm^3^, with the sensitive volume of 1 mm in diameter and 3 mm in length. The scintillation light produced in the active volume of the detector is guided through a 3‐m long optical fiber to a photodiode. Near water equivalency and small dimensions make the W1 PSD suitable for relative dosimetry in small fields, and thus, it was used as the second reference detector for the present study in combination with EBT3 films.^10^, ^44^, ^45^

The scintillator signal is contaminated with Čerenkov radiation, produced in the active volume of the scintillator and in the optical fiber, which needs to be corrected for. The most practical and widely used method for correcting the Čerenkov signal is the spectral discrimination technique which is considered as an accurate and adequate method for removing the Čerenkov signal/light.^46^, ^47^ This method was also adopted in the present study.

In the present study, W1 PSD axis was always oriented parallel to the beam axis. The Čerenkov calibration procedure recommended by the manufacturer, Standard Imaging, for small‐field measurements, based on the method described by Morin et al.^48^ and adopted by others,^10^, ^14^, ^49^, ^50^ was followed in the present study.

The Čerenkov light ratio (CLR) coefficient, needed for correction of the scintillator signal, was calculated as

(4) $$CLR=\frac{M_{max,10}^{Ch1}-M_{min,10}^{Ch1}}{M_{max,10}^{Ch2}-M_{min,10}^{Ch2}}$$

where superscripts *Ch1* and *Ch2* stand for the measured charge *M* with first and second channel, respectively. With the PTW Unidos^*webline*^ electrometer, we measured scintillation signal (green light) in *Ch1*, while for *Ch2* we used the standard PTW Unidos electrometer for measurement of charge mainly produced by Čerenkov radiation (blue light). Subscripts max and min correspond to the maximum (~30 cm) and minimum (~10 cm) fiber length which is in the radiation field, and subscripts 10 stand for the nominal radiation field 10 × 10 cm^2^ applied during the Čerenkov calibration procedure. The Čerenkov‐corrected signal (collected charge) $M_{f_{\mathrm{clin}}}$ for a particular small clinical field $f_{\mathrm{clin}}$ was then obtained from two readings in both channels as

(5) $$M_{f_{\mathrm{clin}}}=M_{f_{\mathrm{clin}}}^{Ch1}-CLR\cdot M_{f_{\mathrm{clin}}}^{Ch2}$$

The CLR coefficient was determined for all four beam energies on both linacs, Elekta Versa HD and Varian TrueBeam. For each beam energy, the energy‐specific values of CLR were obtained from three sets of measurements and the average was used as the final value for CLR. Note that $M_{f_{\mathrm{clin}}}$ in Eq. (5) has the same meaning as $M_{Q_{\mathrm{clin}}}^{f_{\mathrm{clin}}}$ in Eq. (2).

### 2.E. Solid‐state detectors

**Figure 1:**
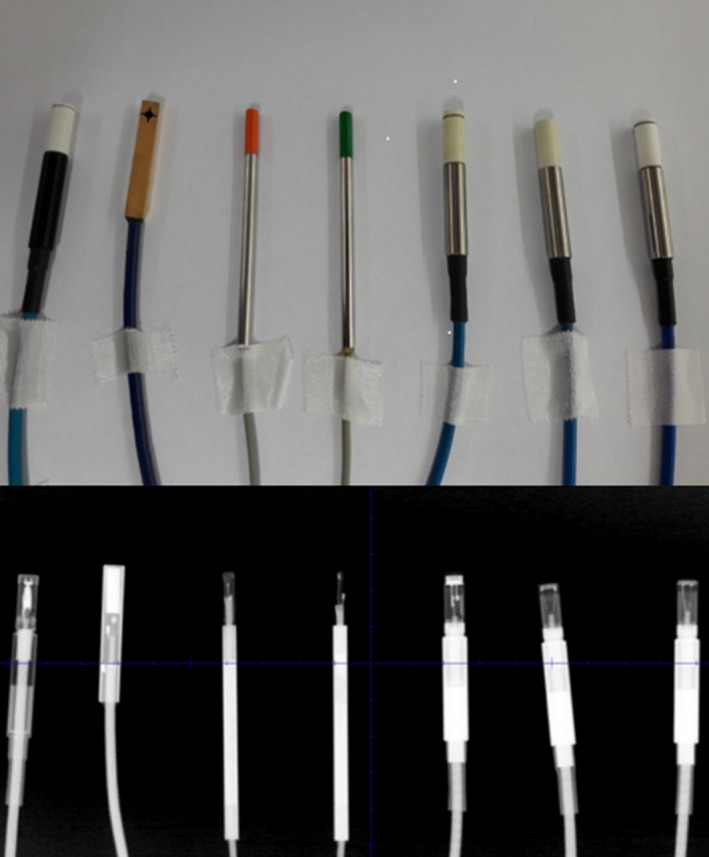
Photo (top) and x‐ray image (bottom) of six diodes and microdiamond detector used in this study. From left to right: PTW 60019 mD, SN EDGE Detector, IBA Razor diode, IBA SFD diode, PTW 60008 Diode P, PTW 60012 Diode E, and PTW 60018 Diode SRS. [Color figure can be viewed at wileyonlinelibrary.com]

Six diodes and a microdiamond detector were selected for the determination of their specific output correction factors: IBA SFD diode and IBA Razor diode (IBA Dosimetry), PTW 60008 Diode P, PTW 60012 Diode E, PTW 60018 Diode SRS, PTW 60019 microDiamond (PTW), and SN EDGE detector (Sun Nuclear, Melbourne, FL, USA). The selection was based on their physical dimensions, characteristics, and availability for clinical use. Figure 1 shows all solid‐state detectors used in the present study while Table 1 lists their basic physical properties and dimensions.

**Table 1:** Summary of basic characteristics and properties of the solid‐state detectors included in this study

Detector type	Active volume dimensions (mm)	Sensitive material	Material density (g/cm^3^)	Z_eff_	Reference depth (mm)
IBA SFD diode	Disk, Ø 0.6 thickness 0.06	Silicone	2.33	14	0.8
IBA Razor diode	Disk, Ø 0.6 thickness 0.02	Silicone	2.33	14	0.8
PTW 60008 Diode P	Disk, Ø 1.2 thickness 0.03	Silicone	2.33	14	2.0
PTW 60012 Diode E	Disk, Ø 1.2 thickness 0.03	Silicone	2.33	14	1.3
PTW 60018 Diode SRS	Disk, Ø 1.2 thickness 0.25	Silicone	2.33	14	1.3
SN EDGE detector	Square 0.8 × 0.8 thickness 0.03	Silicone	2.33	14	0.3
PTW 60019 mD	Disk, Ø 2.2 thickness 0.001	Synthetic diamond	3.53	6	1.0

It is worth mentioning that PTW 60008 P and PTW 60012 E diodes were superseded by newer models, PTW 60016 P and PTW 60017 E diodes respectively; however, the physical construction of the newer models is essentially identical to their predecessors.^19^ The equivalency between the PTW 60008 P and 60016 P diodes and 60012 E and 60017 E diodes has been demonstrated also in the MC study by Francescon et al.,^51^ where the output correction factors were found to be the same.

Before measurements, which were performed with the PTW Unidos^*webline*^ electrometer for all solid‐state detectors, each detector was positioned with its effective point of measurement (physical depth) at the reference depth of 10 cm, and with its stem parallel to the beam axis. The only exception was the SN EDGE detector, which was positioned with its stem orthogonally to the beam axis, due to its different design.

Lateral alignment of detectors along the beam central axis was made in three steps for each detector separately: (a) initial setup using room lasers, (b) repositioning after acquiring lateral beam profiles along cross‐line and in‐line directions, and finally, (c) each detector was moved in manual mode in 0.2 mm steps along both x and y directions and irradiated with 100 MU to find the position where collected charge was maximal. The position where the collected charge reached the highest value was assumed as the central beam axis and final position for each detector. The above procedure for lateral alignment of detectors was done separately for each energy.

For each radiation field, three consecutive measurements of 100 MU each were taken. $M_{Q_{\mathrm{clin}}}^{f_{\mathrm{clin}}}$ in Eq. (2) represents the average value of the three measured values. To limit the influence of environmental conditions, $M_{Q_{\mathrm{ref}}}^{f_{\mathrm{ref}}}$ for the 10 × 10 cm^2^ reference field was always measured prior to the smallest clinical field and at the end of each measurement session for the selected beam energy. The average value of six measurements was then considered as the final value for $M_{Q_{\mathrm{ref}}}^{f_{\mathrm{ref}}}$.

### 2.F. Volume averaging correction

Since EBT3 films and W1 PSD detector are almost water equivalent and have weak energy dependence, it is assumed in the present study that they have no perturbation correction factors.^1^ However, their signals still need to be corrected for volume averaging effects for the determination of field output factors. It is evident for scintillator as it has a finite size. However, it might not be so evident for radiochromic films. The radiochromic film is considered as a detector with almost infinite resolution. Inherently, it might be the case. However, there are two limitations which need to be considered in clinical dosimetry — scanning resolution, which the user decides upon and the selected size of the film detector. Both need to be defined, that is, one needs to define a specific finite area (ROI) of the film which will be used as a detector area of the EBT3 film for data analysis as well as scanning resolution which will be used for subsequent evaluation. As mentioned earlier, in this study, the ROI was chosen to have a diameter of 0.5 mm, and the scanning resolution was chosen to have a value of 150 dpi for field output factor measurements.

According to TRS‐483, $k_{Q_{\mathrm{clin}},Q_{\mathrm{ref}}}^{f_{\mathrm{clin}},f_{\mathrm{ref}}}$ for these two detectors can be simplified as follows

(6) $$k_{Q_{\mathrm{clin}},Q_{\mathrm{ref}}}^{f_{\mathrm{clin}},f_{\mathrm{ref}}}=k_{\mathrm{vol}}$$

where $k_{\mathrm{vol}}$ is the volume averaging correction factor due to the detector's finite size and represents the only correction factor that was considered for the two reference detectors in this study and has a purely geometrical concept.

Combining Eqs. (2) and (6), average signal readings $M_{Q_{\mathrm{clin}}}^{f_{\mathrm{clin}}}$ of EBT3 films and W1 PSD can be corrected for volume averaging $k_{\mathrm{vol}}$ to determine discrete field output factors for small clinical fields as

(7) $$\Omega_{Q_{\mathrm{clin}},Q_{\mathrm{ref}}}^{f_{\mathrm{clin}},f_{\mathrm{ref}}}=\frac{M_{Q_{\mathrm{clin}}}^{f_{\mathrm{clin}}}}{M_{Q_{\mathrm{ref}}}^{f_{\mathrm{ref}}}}k_{\mathrm{vol}}$$

Doses measured from EBT3 films were employed to calculate $k_{\mathrm{vol}}$ factors. For each small field $S_{\mathrm{clin}}$ and beam energy E, film doses in a region of interest with dimensions 3 mm × 3 mm (i.e., from −1.5 to +1.5 mm in cross‐plane direction x and in‐plane direction y) centered on the beam axes were fitted to a bivariate Gaussian function

(8) $$f\left(x,y,S_{\mathrm{clin}},E\right)=a\cdot e^{-\frac{1}{2}\left({\left(\frac{x}{b}\right)}^{2}+{\left(\frac{y}{c}\right)}^{2}\right)}$$

using fit parameters, a, b, and c. The volume averaging correction factors $k_{\mathrm{vol}}$ were calculated as

(9) $$k_{\mathrm{vol}}=\frac{a\cdot \pi r^{2}}{a\cdot \int \int_{A}f\left(x,y,S_{\mathrm{clin}},E\right)dxdy}$$

This is similar to the previously published studies by Morin et al.^48^ and Papaconstadopoulos et al.^10^, ^34^ *r* in Eq. (9) is the radius of the detector's sensitive volume in a plane orthogonal to the beam axis. The detector's radius *r* was replaced with the size of the equivalent square field side length d (note that this “equivalent square field size length d” is defined here for performing the integration of Eq. (9); this is different from the “equivalent square small field size $S_{\mathrm{clin}}$” defined in Eq. (3)), applying the expression $\pi r^{2}=d^{2}$, yields the result

(10) $$k_{\mathrm{vol}}=\frac{d^{2}}{2\pi bc\cdot erf\left(\frac{d/2}{\sqrt{2}\cdot b}\right)erf\left(\frac{d/2}{\sqrt{2}\cdot c}\right)}$$

Therefore, given the field size $S_{\mathrm{clin}}$ and beam energy E, the parameters d, b, and c are necessary to calculate the $k_{\mathrm{vol}}$ factor of a specific detector. The first parameter d was obtained from the detector specifications, with the exception of EBT3 films, where the diameter of the detector (i.e., central ROI for the EBT3 films) was chosen as 0.5 mm. For W1 PSD, we used the value r = 0.5 mm following vendor specifications. The other two parameters were derived from film dose fit. $k_{\mathrm{vol}}$ were calculated for all solid‐state detectors including the EBT3 and W1 PSD using the expression given in Eq. (10).

To check whether 150 dpi scanning resolution was adequate for the determination of $k_{\mathrm{vol}}$, several sets of films were scanned for the smallest field sizes using the 1200 dpi resolution and the values of $k_{\mathrm{vol}}$ thus obtained were compared with those obtained using the 150 dpi scanning resolution. No differences in $k_{\mathrm{vol}}$ were observed between both approaches; therefore, 150 dpi scanning resolution was used for film scanning throughout this study.

### 2.G. Field output factors

For the determination of field output factors, EBT3 films and W1 PSD were used equivalently, without any preference.Signals $M_{Q_{\mathrm{clin}}}^{f_{\mathrm{clin}}}\left(EBT3\right)$ measured with EBT3 films were corrected for volume averaging as

(11) $$M_{Q_{\mathrm{clin}}}^{f_{\mathrm{clin}}}{\left(EBT3\right)}_{\mathrm{corr}}=M_{Q_{\mathrm{clin}}}^{f_{\mathrm{clin}}}\left(EBT3\right)\cdot k_{\mathrm{vol}}^{f_{\mathrm{clin}}}\left(EBT3\right)$$

where $k_{\mathrm{vol}}^{f_{\mathrm{clin}}}$denotes the volume averaging correction factor for the specific clinical field $f_{\mathrm{clin}}$ (in this study, $f_{\mathrm{clin}}\equiv S_{\mathrm{clin}}$). In addition to volume averaging correction, signals for the W1 PSD detector, $M_{Q_{\mathrm{clin}}}^{f_{\mathrm{clin}}}\left(W1PSD\right)$, were further normalized as

(12) $$M_{Q_{\mathrm{clin}}}^{f_{\mathrm{clin}}}{\left(W1PSD\right)}_{N,corr}=\frac{M_{Q_{\mathrm{clin}}}^{f_{\mathrm{clin}}}\left(W1PSD\right)\cdot k_{\mathrm{vol}}^{f_{\mathrm{clin}}}\left(W1PSD\right)}{\overset{¯}{\epsilon }}$$

where $\overset{¯}{\epsilon }$ is the average value of ratios of measured signals between W1 PSD and EBT3 films for each particular beam energy of the specific linac, calculated as

(13) $$\overset{¯}{\epsilon }=\frac{1}{9}\sum_{f_{\mathrm{clin}}}\frac{M_{Q_{\mathrm{clin}}}^{f_{\mathrm{clin}}}\left(W1PSD\right)\cdot k_{\mathrm{vol}}^{f_{\mathrm{clin}}}\left(W1PSD\right)}{M_{Q_{\mathrm{clin}}}^{f_{\mathrm{clin}}}\left(EBT3\right)\cdot k_{\mathrm{vol}}^{f_{\mathrm{clin}}}\left(EBT3\right)}$$

where summation goes over all nine clinical small fields selected for this study.

$M_{Q_{\mathrm{clin}}}^{f_{\mathrm{clin}}}{\left(EBT3\right)}_{\mathrm{corr}}$ values obtained from Eq. (11) determined by EBT3 films and $M_{Q_{\mathrm{clin}}}^{f_{\mathrm{clin}}}{\left(W1PSD\right)}_{N,corr}$ values from Eq. (12) determined by W1 PSD, were fitted together by the analytical function proposed by Sauer and Wilbert,^8^

(14) $$\Omega \left(S_{\mathrm{clin}}\right)=P_{\infty }\frac{S_{\mathrm{clin}}^{n}}{l^{n}+S_{\mathrm{clin}}^{n}}+S_{\infty }\left(1-e^{-b\cdot S_{\mathrm{clin}}}\right)$$

which was normalized to $\Omega \left(S_{\mathrm{clin}}=10\;\text{cm}\right)=1$. $P_{\infty }$, $S_{\infty }$, l, n and b are the fitting parameters, adjusted according to a routine, which optimizes the maximum likelihood estimation (MLE). For brevity and to avoid potential ambiguity, subscripts and superscripts were omitted from $\Omega (S_{\mathrm{clin}})$, which is in the form of an analytical function, unlike the $\Omega_{Q_{\mathrm{clin}},Q_{\mathrm{ref}}}^{f_{\mathrm{clin}},f_{\mathrm{ref}}}$ which is used for discrete values of field output factors. Furthermore, $S_{\mathrm{clin}}$ was kept in the same form as in Eq. (3) to emphasize that it stands for the equivalent square small field sizes rather than the nominal field sizes; it has the same meaning as *s*, used by Sauer and Wilbert in their original work. The use of analytical function, instead of the discrete values for field output factors, reduces uncertainties in W1 PSD and EBT3 film measurements. In addition, the functional form of field output factors $\Omega (S_{\mathrm{clin}})$ allows one to calculate discrete values for field output factors for any equivalent square small field size within the range of small field sizes used in this study.

### 2.H. Output correction factors

For every solid‐state detector and for each measured equivalent square small field size $S_{\mathrm{clin}}$, discrete values of detector‐specific output correction factors $k_{Q_{\mathrm{clin}},Q_{\mathrm{ref}}}^{f_{\mathrm{clin}},f_{\mathrm{ref}}}\left(S_{\mathrm{clin}}\right)$ were calculated as

(15) $$k_{Q_{\mathrm{clin}},Q_{\mathrm{ref}}}^{f_{\mathrm{clin}},f_{\mathrm{ref}}}\left(S_{\mathrm{clin}}\right)=\frac{\Omega_{Q_{\mathrm{clin}},Q_{\mathrm{ref}}}^{f_{\mathrm{clin}},f_{\mathrm{ref}}}}{\frac{M_{Q_{\mathrm{clin}}}^{f_{\mathrm{clin}}}}{M_{Q_{\mathrm{ref}}}^{f_{\mathrm{ref}}}}}$$

Discrete values of field output factors $\Omega_{Q_{\mathrm{clin}},Q_{\mathrm{ref}}}^{f_{\mathrm{clin}},f_{\mathrm{ref}}}$ were obtained from the analytical function $\Omega (S_{\mathrm{clin}})$ in Eq. (14). $k_{Q_{\mathrm{clin}},Q_{\mathrm{ref}}}^{f_{\mathrm{clin}},f_{\mathrm{ref}}}\left(S_{\mathrm{clin}}\right)$ values were fitted by the analytical function published in TRS‐483^1^

(16) $$k\left(S_{\mathrm{clin}}\right)=\frac{1+d\cdot e^{-}\frac{10-a}{b}}{1+d\cdot e^{-}\frac{S_{\mathrm{clin}}-a}{b}}+c\cdot \left(S_{\mathrm{clin}}-10\right)$$

with fitting coefficients, a, b, and c. Instead of symbol S, which is used in TRS‐483 in the analytical function in Eq. (16), symbol $S_{\mathrm{clin}}$ was used in this study to emphasize that in this study equivalent square field sizes were used without exception. As in the case for field output factors, subscripts and superscripts are omitted in the notations for output correction factors in Eq. (16) to indicate that in this case, output correction factors have functional form.

Also, discrete values of $k_{Q_{\mathrm{clin}},Q_{\mathrm{ref}}}^{f_{\mathrm{clin}},f_{\mathrm{ref}}}\left(S_{\mathrm{clin}}\right)$ were calculated and reported for all solid‐state detectors, energies and small fields, applying Eq. (15).

## 3 Results

### 3.A. Equivalent square small field size $S_{\mathrm{clin}}$

For each nominal field size, corresponding equivalent square field sizes $S_{\mathrm{clin}}$ were calculated based on EBT3 film measurements and applying Eq. (3). Data for nominal and equivalent square field sizes are presented in Table 2. Throughout the paper, field sizes will be indicated with nominal values; however, they will represent without exception the corresponding $S_{\mathrm{clin}}$ values. For brevity, square field sizes will be denoted as nominal square field side lengths, for example, instead of 0.5 × 0.5 cm^2^, notation 0.5 cm will be used in the rest of the paper.

**Table 2:** Nominal field sizes and corresponding equivalent square small field sizes $S_{\mathrm{clin}}$ on the Elekta Versa HD and Varian TrueBeam linacs measured with EBT3 films and applying Eq. (3)

Nominal square field side length (cm)	$S_{\mathrm{clin}}\left(\text{cm}\right)$ — Elekta Versa HD				$S_{\mathrm{clin}}\left(\text{cm}\right)$ — Varian TrueBeam			
	6 MV WFF	6 MV FFF	10 MV WFF	10 MV FFF	6 MV WFF	6 MV FFF	10 MV WFF	10 MV FFF
0.5	0.60	0.59	0.62	0.58	0.56	0.54	0.57	0.55
0.8	0.87	0.85	0.87	0.86	0.81	0.82	0.84	0.81
1.0	1.03	1.03	1.06	1.04	1.01	0.99	1.03	1.02
1.5	1.51	1.52	1.55	1.52	1.50	1.49	1.52	1.51
2.0	2.04	2.03	2.05	2.04	2.00	1.99	2.01	1.99
3.0	3.06	3.04	3.08	3.02	3.03	3.00	3.00	2.98
4.0	4.04	4.03	4.06	4.01	4.03	3.99	4.02	3.98
5.0	5.04	5.01	5.05	4.99	5.02	5.00	5.01	4.96
10.0	10.04	9.94	10.05	9.90	10.03	9.96	10.02	9.87

While equivalent square small field sizes $S_{\mathrm{clin}}$ are nearly identical to the nominal field sizes for field sizes ≥1 cm, they differ significantly for the two smallest fields, regardless of the energy or collimation (linac) being used.

Uncertainty of field size dimensions was determined from EBT3 films and was found to be <0.1 mm. The same level of uncertainty of field setup reproducibility was determined with repeated measurements of lateral beam profiles with diodes at FWHM for smallest field sizes.

### 3.B. Field output factors

**Figure 2:**
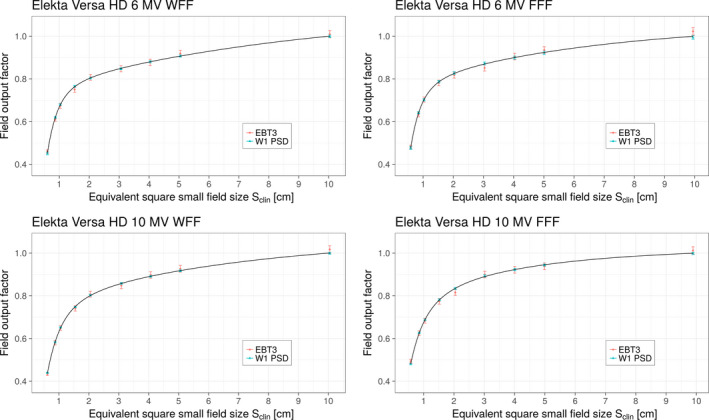
Field output factors vs $S_{\mathrm{clin}}$ on the Elekta Versa HD linac for four investigated beam energies. The red and green symbols represent field output factors along with their respective uncertainties (1 SD) determined using EBT3 film and W1 PSD detectors, respectively. The solid lines represent fits to both sets of data using the analytic function given in Eq. (14). [Color figure can be viewed at wileyonlinelibrary.com]

Figure 2 shows the field output factors determined from measurements made on the Elekta Versa HD linac with EBT3 films and W1 PSD detectors. The red circles and blue triangles represent field output factors for the EBT3 film and W1 PSD detectors, respectively. Measured signals were corrected for volume averaging using Eqs. (11) and (12) when $k_{\mathrm{vol}}$ exceeded 0.1%; for the Elekta linac, these values are given in Table 3 for all beam energies. The uncertainties for each data point correspond to 1 SD. The solid lines in Fig. 2 represent fits to both sets of data using the analytical function given in Eq. (14). Relative uncertainties (1 SD) of the fits were largest for the smallest field size of 0.5 cm and were found to be 1.0%, 1.3%, 1.2%, and 1.4% for beam energies 6 MV WFF, 6 MV FFF, 10 MV WFF, and 10 MV FFF respectively. In addition to the curves in Fig. 2, discrete values of field output factors $\Omega_{Q_{\mathrm{clin}},Q_{\mathrm{ref}}}^{f_{\mathrm{clin}},f_{\mathrm{ref}}}$ were calculated using Eq. (15) and are given in Table 4 for all selected small fields and energies for the Elekta Versa HD and Varian TrueBeam linacs.

**Table 3:** Calculated volume averaging correction factors $k_{\mathrm{vol}}$ for EBT3 films and W1 PSD for three smallest field sizes on the Elekta Versa HD linac which were taken into account for the determination of field output factors

Field size^a^ (cm)	EBT3	W1 PSD	EBT3	W1 PSD
	6 MV WFF		6 MV FFF	
0.5	1.002	1.008	1.002	1.008
0.8	1.000	1.002	1.000	1.002
1.0	1.000	1.001	1.000	1.001
	10 MV WFF		10 MV FFF	
0.5	1.002	1.007	1.002	1.007
0.8	1.000	1.002	1.000	1.002
1.0	1.000	1.001	1.000	1.001

a Field size is indicated as the nominal square field side length. The relationship between the nominal field size and the corresponding equivalent square small field sizes $S_{\mathrm{clin}}$ is provided in Table 2 for all energies and field sizes.

**Table 4:** Discrete values of field output factors for nine selected field sizes calculated from the analytical function in Eq. (14) for all investigated beam energies on two different linacs. Uncertainties (1 SD) are shown in brackets and represent absolute uncertainties in the last one or two digits

Field size^a^ (cm)	Elekta Versa HD				Varian TrueBeam			
	6 MV WFF	6 MV FFF	10 MV WFF	10 MV FFF	6 MV WFF	6 MV FFF	10 MV WFF	10 MV FFF
0.5	0.454 (5)	0.478 (6)	0.438 (5)	0.481 (7)	0.482 (9)	0.513 (11)	0.412 (10)	0.468 (7)
0.8	0.620 (5)	0.640 (6)	0.584 (5)	0.626 (6)	0.632 (8)	0.654 (10)	0.556 (8)	0.615 (7)
1.0	0.678 (5)	0.703 (6)	0.650 (5)	0.688 (6)	0.694 (8)	0.701 (10)	0.625 (9)	0.687 (7)
1.5	0.763 (5)	0.786 (6)	0.750 (5)	0.780 (6)	0.761 (9)	0.772 (11)	0.730 (9)	0.780 (8)
2.0	0.804 (5)	0.825 (6)	0.801 (6)	0.835 (6)	0.793 (9)	0.806 (11)	0.785 (10)	0.828 (8)
3.0	0.849 (5)	0.870 (6)	0.858 (6)	0.890 (6)	0.834 (9)	0.844 (11)	0.844 (10)	0.881 (8)
4.0	0.880 (5)	0.900 (7)	0.891 (6)	0.923 (7)	0.866 (9)	0.871 (11)	0.880 (10)	0.914 (9)
5.0	0.907 (5)	0.924 (7)	0.918 (6)	0.945 (7)	0.893 (9)	0.895 (10)	0.907 (10)	0.937 (9)
10.0	1.001 (0)	0.999 (0)	1.001 (0)	0.999 (0)	1.001 (0)	0.999 (0)	1.000 (0)	0.999 (0)

a Field size is indicated as the nominal square field side length. The relationship between the nominal field size and the corresponding equivalent square small field sizes $S_{\mathrm{clin}}$ is provided in Table 2 for all energies and field sizes. Note that because $S_{\mathrm{clin}}$ are not exactly equal to 10.0 cm (Table 2), corresponding field output factors differ from value 1.000.

**Figure 3:**
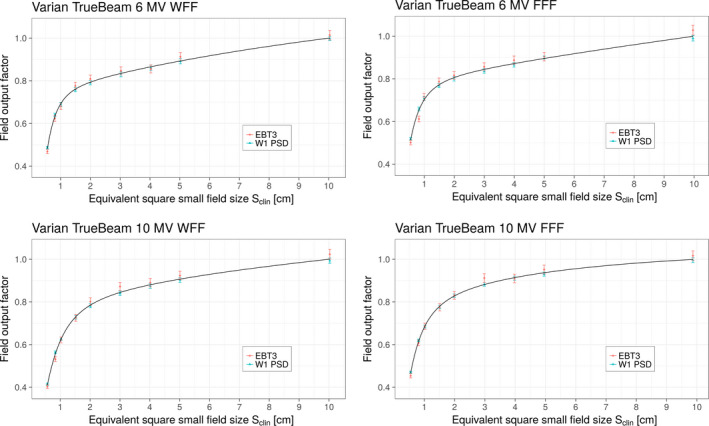
Field output factors vs $S_{\mathrm{clin}}$on the Varian TrueBeam linac for four investigated beam energies. The red and blue symbols represent field output factors along with their respective uncertainties (1 SD) determined using EBT3 film and W1 PSD detectors, respectively. The solid lines represent fits to both sets of data using the analytic function given in Eq. (14). [Color figure can be viewed at wileyonlinelibrary.com]

Field output factors determined from measurements made on the Varian TrueBeam linac with EBT3 films and W1 PSD detectors are shown in Fig. 3. The red circles and blue triangles represent field output factors for the EBT3 film and W1 PSD detectors, respectively. Similar to the Elekta linac, measured signals were corrected for volume averaging using Eqs. (11) and (12) when $k_{\mathrm{vol}}$ exceeded 0.1%; for the Varian linac values of $k_{\mathrm{vol}}$ are given in Table 5 for all beam energies. The solid lines in Fig. 3 represent fits to both sets of data using the analytical function given by Eq. (14). The largest relative uncertainties (1 SD) of the fits were again found for the smallest field size of 0.5 cm and were found to be 2.0%, 2.2%, 2.5%, and 1.6% for beam energies 6 MV WFF, 6 MV FFF, 10 MV WFF, and 10 MV FFF, respectively. Discrete values of field output factors for all selected small fields and energies for the Varian TrueBeam are given in Table 4. These values were calculated using the fitting function given by Eq. (14).

**Table 5:** Calculated volume averaging correction factors $k_{\mathrm{vol}}$ for EBT3 films and W1 PSD for the three smallest field sizes on the Varian TrueBeam linac; these values were taken into account for the determination of field output factors

Field size^a^ (cm)	EBT3	W1 PSD	EBT3	W1 PSD
	6 MV WFF		6 MV FFF	
0.5	1.002	1.009	1.002	1.009
0.8	1.000	1.004	1.000	1.002
1.0	1.000	1.001	1.000	1.000
	10 MV WFF		10 MV FFF	
0.5	1.002	1.008	1.002	1.008
0.8	1.001	1.004	1.000	1.003
1.0	1.000	1.001	1.000	1.001

a Field size is indicated as the nominal square field side length. The relationship between the nominal field size and the corresponding equivalent square small field sizes $S_{\mathrm{clin}}$ is provided in Table 2 for all energies and field sizes.

The five fitting coefficients of the analytical function from Eq. (14) are given Table 6 for all investigated beam energies on both linacs.

**Table 6:** Values of fitting parameters for the analytical function given in Eq. (14). This function was used to fit the field output factor datasets obtained using the EBT3 films and W1 PSD detectors on the two linacs for four beam energies

*E*	$P_{\infty }$	n	l	$S_{\infty }$	*b*
	Elekta Versa HD				
6 MV WFF	0.751	2.701	0.542	0.384	0.105
6 MV FFF	0.767	2.614	0.514	0.299	0.151
10 MV WFF	0.774	2.183	0.578	0.313	0.130
10 MV FFF	0.829	1.791	0.511	0.198	0.214
	Varian TrueBeam				
6 MV WFF	0.741	2.646	0.461	0.508	0.072
6 MV FFF	0.790	2.097	0.419	1.424	0.016
10 MV WFF	0.816	1.844	0.588	0.478	0.050
10 MV FFF	0.816	1.904	0.497	0.227	0.173

To quantify statistical significance of differences between field output factors given in Table 4 for WFF and FFF beams for a particular energy and linac, a one‐tailed Student's *t*‐test was performed. These result are shown in Table 7.

**Table 7:** Statistical significance (*P*‐values) of differences in field output factors between WFF and FFF beams on the Elekta Versa HD and Varian TrueBeam linacs for 6 and 10 MV beams. For the determination of *P*‐values, a one‐tailed Student's *t*‐test was performed

*P*‐values				
Field size^a^ (cm)	Elekta Versa HD		Varian TrueBeam	
	6 MV WFF/FFF	10 MV WFF/FFF	6 MV WFF/FFF	10 MV WFF/FFF
0.5	0.023	0.010	0.049	0.012
0.8	0.034	0.009	0.069	0.008
1.0	0.023	0.010	0.263	0.009
1.5	0.026	0.015	0.189	0.014
2.0	0.034	0.014	0.166	0.022
3.0	0.033	0.016	0.213	0.030
4.0	0.039	0.019	0.333	0.035
5.0	0.058	0.028	0.427	0.043
10.0	0.006	0.015	0.001	0.038

a Field size is indicated as the nominal square field side length. The relationship between the nominal field size and the corresponding equivalent square small field sizes $S_{\mathrm{clin}}$ is provided in Table 2 for all energies and field sizes.

### 3.C. Detector‐specific output correction factors

**Figure 4:**
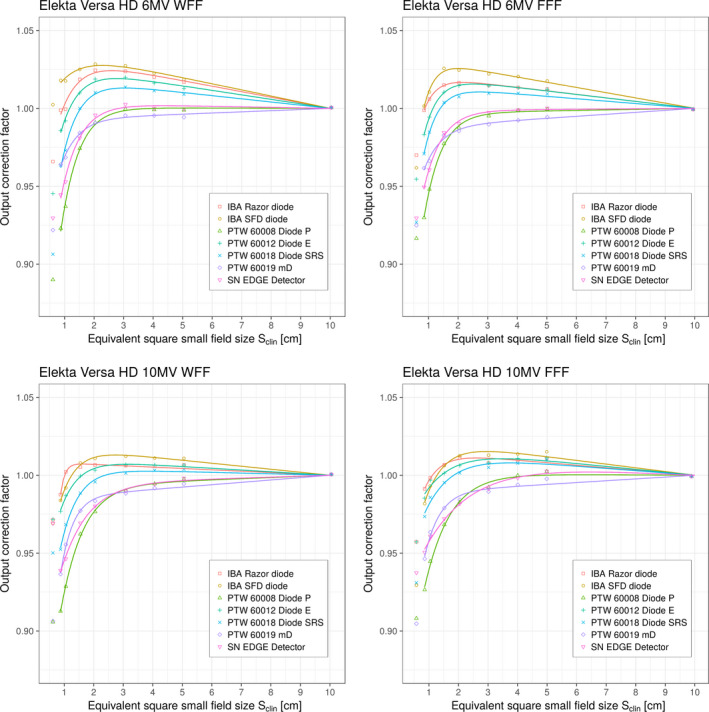
Detector‐specific output correction factors $k_{Q_{\mathrm{clin}},Q_{\mathrm{ref}}}^{f_{\mathrm{clin}},f_{\mathrm{ref}}}$ for seven solid‐state detectors for four beam energies on the Elekta Versa HD linac. Output correction factors are presented as individual values/points and as analytical function applying Eqs. (15) and (16), respectively. Measured data represent “total” correction factors and include contributions from both volume averaging effect as well as perturbation correction factors. 0.5 cm field was not considered for fitting. [Color figure can be viewed at wileyonlinelibrary.com]

Figure 4 shows detector‐specific output correction factors $k_{Q_{\mathrm{clin}},Q_{\mathrm{ref}}}^{f_{\mathrm{clin}},f_{\mathrm{ref}}}\left(S_{\mathrm{clin}}\right)$as a function of equivalent square small field sizes $S_{\mathrm{clin}}$ for seven solid‐state detectors and four beam energies; these were determined using Eq. (15) on the Elekta Versa HD linac. The solid curves in these figures represent fits to the data points using the analytic function $k(S_{\mathrm{clin}})$ given by Eq. (16). For brevity $k_{Q_{\mathrm{clin}},Q_{\mathrm{ref}}}^{f_{\mathrm{clin}},f_{\mathrm{ref}}}\left(S_{\mathrm{clin}}\right)$ will be denoted as $k_{Q_{\mathrm{clin}},Q_{\mathrm{ref}}}^{f_{\mathrm{clin}},f_{\mathrm{ref}}}$ henceforth. The data points obtained from Eq. (15) were fitted down to the field size of 0.8 cm to ensure acceptable fit of the selected fitting function. For comparison and further analysis, individual discrete values for $k_{Q_{\mathrm{clin}},Q_{\mathrm{ref}}}^{f_{\mathrm{clin}},f_{\mathrm{ref}}}$, obtained for the Elekta Versa HD linac using Eq. (15), are provided in Table 8 for all energies and selected field sizes. It should be noted that the $k_{Q_{\mathrm{clin}},Q_{\mathrm{ref}}}^{f_{\mathrm{clin}},f_{\mathrm{ref}}}$ correction factor thus determined represents the “total” correction factor for a particular detector and includes contributions for both volume averaging correction factor as well as perturbations correction factors. The $k_{Q_{\mathrm{clin}},Q_{\mathrm{ref}}}^{f_{\mathrm{clin}},f_{\mathrm{ref}}}$ factors thus determined can then be compared directly with those reported in TRS‐483 for the corresponding detectors.

**Table 8:** Detector‐specific output correction factors $k_{Q_{\mathrm{clin}},Q_{\mathrm{ref}}}^{f_{\mathrm{clin}},f_{\mathrm{ref}}}$ obtained on the Elekta Versa HD linac for six diodes and a microdiamond detector and four investigated beam energies. These values were obtained by using Eq. (15). Values in brackets show absolute uncertainties (1 SD) in the last one or two digits. Measured data represent “total” correction factors and include contributions from both volume averaging effect as well as perturbation correction factors

*E*	Field size^a^ (cm)	IBA SFD diode	IBA Razor diode	PTW 60008 Diode P	PTW 60012 Diode E	PTW 60018 Diode SRS	SN EDGE Detector	PTW 60019 mD
6 MV WFF	0.5	1.002 (10)	0.966 (10)	0.890 (9)	0.945 (10)	0.906 (9)	0.930 (10)	0.922 (10)
	0.8	1.018 (8)	0.999 (8)	0.923 (7)	0.986 (7)	0.964 (7)	0.945 (7)	0.964 (8)
	1.0	1.018 (7)	0.999 (7)	0.937 (6)	0.992 (7)	0.973 (7)	0.953 (7)	0.968 (7)
	1.5	1.025 (6)	1.019 (7)	0.974 (6)	1.010 (6)	1.000 (6)	0.981 (6)	0.984 (7)
	2.0	1.028 (7)	1.024 (7)	0.991 (6)	1.019 (7)	1.010 (6)	0.996 (7)	0.991 (7)
	3.0	1.027 (6)	1.024 (6)	1.000 (6)	1.020 (6)	1.014 (6)	1.003 (6)	0.995 (6)
	4.0	1.023 (6)	1.020 (6)	0.999 (6)	1.016 (6)	1.011 (6)	1.000 (6)	0.995 (6)
	5.0	1.019 (6)	1.017 (6)	0.999 (6)	1.013 (6)	1.009 (6)	1.000 (6)	0.994 (6)
	10.0	1.001 (0)	1.001 (2)	1.001 (1)	1.001 (0)	1.001 (0)	1.001 (2)	1.001 (3)
6 MV FFF	0.5	0.962 (12)	0.970 (12)	0.916 (12)	0.955 (12)	0.927 (12)	0.930 (12)	0.925 (12)
	0.8	1.001 (9)	0.999 (9)	0.930 (9)	0.983 (9)	0.971 (9)	0.949 (9)	0.962 (9)
	1.0	1.011 (9)	1.006 (9)	0.948 (8)	0.994 (8)	0.985 (8)	0.961 (8)	0.966 (8)
	1.5	1.026 (8)	1.015 (8)	0.977 (7)	1.010 (8)	1.004 (8)	0.984 (8)	0.983 (8)
	2.0	1.025 (8)	1.016 (8)	0.987 (8)	1.015 (8)	1.007 (8)	0.990 (8)	0.985 (8)
	3.0	1.022 (7)	1.015 (7)	0.995 (7)	1.014 (7)	1.010 (7)	0.997 (7)	0.990 (7)
	4.0	1.020 (8)	1.013 (7)	0.999 (7)	1.013 (7)	1.010 (7)	0.999 (7)	0.992 (7)
	5.0	1.018 (8)	1.012 (8)	1.000 (8)	1.012 (8)	1.009 (8)	1.000 (8)	0.994 (8)
	10.0	0.999 (0)	0.999 (0)	0.999 (1)	0.999 (1)	0.999 (0)	0.999 (1)	0.999 (0)
10 MV WFF	0.5	0.969 (12)	0.971 (12)	0.906 (11)	0.972 (12)	0.950 (12)	0.969 (12)	0.906 (11)
	0.8	0.984 (8)	0.988 (8)	0.913 (8)	0.977 (8)	0.952 (8)	0.939 (8)	0.936 (8)
	1.0	0.992 (8)	1.002 (8)	0.929 (7)	0.987 (8)	0.968 (8)	0.946 (7)	0.955 (8)
	1.5	1.008 (7)	1.005 (7)	0.962 (7)	0.999 (7)	0.989 (7)	0.969 (7)	0.977 (7)
	2.0	1.011 (7)	1.007 (7)	0.977 (7)	1.003 (7)	0.996 (7)	0.980 (7)	0.984 (7)
	3.0	1.012 (7)	1.006 (7)	0.990 (6)	1.007 (6)	1.001 (7)	0.990 (7)	0.988 (7)
	4.0	1.011 (7)	1.006 (7)	0.995 (6)	1.007 (6)	1.003 (7)	0.994 (6)	0.992 (7)
	5.0	1.011 (7)	1.007 (7)	0.997 (7)	1.007 (7)	1.004 (7)	0.998 (7)	0.995 (7)
	10.0	1.001 (0)	1.001 (2)	1.001 (1)	1.001 (1)	1.001 (2)	1.001 (1)	1.001 (3)
10 MV FFF	0.5	0.929 (13)	0.957 (13)	0.908 (13)	0.957 (13)	0.931 (13)	0.937 (13)	0.905 (13)
	0.8	0.982 (10)	0.991 (10)	0.926 (9)	0.985 (10)	0.973 (10)	0.951 (9)	0.946 (9)
	1.0	0.993 (9)	0.998 (9)	0.945 (8)	0.997 (9)	0.986 (9)	0.961 (9)	0.963 (9)
	1.5	1.006 (8)	1.007 (8)	0.968 (7)	1.001 (8)	0.995 (8)	0.972 (7)	0.979 (7)
	2.0	1.012 (8)	1.011 (8)	0.983 (7)	1.006 (8)	1.001 (8)	0.982 (8)	0.984 (8)
	3.0	1.013 (7)	1.008 (7)	0.992 (7)	1.008 (7)	1.005 (7)	0.991 (7)	0.990 (7)
	4.0	1.014 (8)	1.010 (8)	1.000 (7)	1.011 (8)	1.008 (8)	0.998 (7)	0.994 (7)
	5.0	1.015 (8)	1.011 (8)	1.002 (8)	1.011 (8)	1.010 (8)	1.003 (8)	0.998 (8)
	10.0	0.999 (1)	0.999 (1)	0.999 (0)	0.999 (2)	0.999 (2)	0.999 (1)	0.999 (1)

Field size is indicated as the nominal square field side length. The relationship between the nominal field size and the corresponding equivalent square small field sizes $S_{\mathrm{clin}}$ is provided in Table 2 for all energies and field sizes.

**Figure 5:**
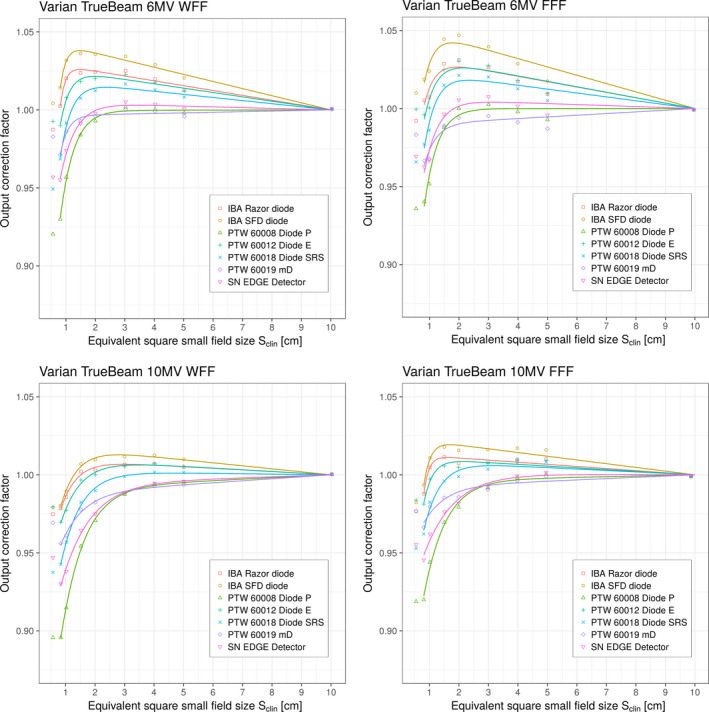
Detector‐specific output correction factors $k_{Q_{\mathrm{clin}},Q_{\mathrm{ref}}}^{f_{\mathrm{clin}},f_{\mathrm{ref}}}$ for seven solid‐state detectors for four beam energies on the Varian TrueBeam linac. Output correction factors are presented as individual values/points and as analytical function applying Eqs. (15) and (16), respectively. Measured data represent “total” correction factors and include contributions from both volume averaging effect as well as perturbation correction factors. 0.5 cm field was not considered for fitting. [Color figure can be viewed at wileyonlinelibrary.com]

Figure 5 shows detector‐specific output correction factors $k_{Q_{\mathrm{clin}},Q_{\mathrm{ref}}}^{f_{\mathrm{clin}},f_{\mathrm{ref}}}$ for seven solid‐state detectors for four beam energies on the Varian TrueBeam linac. Output correction factors are presented as individual values/points and as analytical function applying Eqs. (15) and (16), respectively. The data obtained from Eq. (15) were fitted down to the field size of 0.8 cm to ensure acceptable fit of the selected fitting function. For comparison and further analysis, individual discrete values for $k_{Q_{\mathrm{clin}},Q_{\mathrm{ref}}}^{f_{\mathrm{clin}},f_{\mathrm{ref}}}$ are provided in Table 9 for all energies and field sizes. $k_{Q_{\mathrm{clin}},Q_{\mathrm{ref}}}^{f_{\mathrm{clin}},f_{\mathrm{ref}}}$ represent “total” output correction factors for a particular detector and include contributions from both volume averaging effect as well as perturbation correction factors.

**Table 9:** Detector‐specific output correction factors $k_{Q_{\mathrm{clin}},Q_{\mathrm{ref}}}^{f_{\mathrm{clin}},f_{\mathrm{ref}}}$ obtained on the Varian TrueBeam linac for six diodes and microdiamond detector and four investigated beam energies. These values were obtained by using Eq. (15). Values in brackets show absolute uncertainties (1 SD) in the last one or two digits. Measured data represent “total” correction factors and include contributions from both volume averaging effect as well as perturbation correction factors

*E*	Field size^a^ (cm)	IBA SFD diode	IBA Razor diode	PTW 60008 Diode P	PTW 60012 Diode E	PTW 60018 Diode SRS	SN EDGE Detector	PTW 60019 mD
6 MV WFF	0.5	1.004 (20)	0.987 (19)	0.920 (18)	0.993 (20)	0.949 (19)	0.957 (19)	0.983 (19)
	0.8	1.014 (13)	1.002 (13)	0.930 (12)	0.990 (13)	0.969 (13)	0.955 (13)	0.971 (13)
	1.0	1.032 (12)	1.020 (12)	0.957 (11)	1.008 (12)	0.991 (12)	0.974 (12)	0.986 (12)
	1.5	1.036 (12)	1.024 (11)	0.984 (11)	1.018 (11)	1.007 (11)	0.991 (11)	0.993 (11)
	2.0	1.036 (12)	1.024 (12)	0.993 (11)	1.020 (12)	1.012 (12)	0.998 (11)	0.996 (11)
	3.0	1.034 (11)	1.025 (11)	1.002 (11)	1.023 (11)	1.017 (11)	1.005 (11)	1.000 (11)
	4.0	1.029 (11)	1.020 (11)	1.000 (11)	1.017 (11)	1.013 (11)	1.004 (11)	0.999 (11)
	5.0	1.021 (11)	1.013 (11)	0.998 (10)	1.012 (11)	1.008 (10)	1.001 (10)	0.996 (10)
	10.0	1.001 (1)	1.001 (0)	1.001 (1)	1.001 (1)	1.001 (1)	1.001 (1)	1.001 (0)
6 MV FFF	0.5	1.010 (23)	0.992 (22)	0.936 (21)	1.000 (22)	0.966 (22)	0.969 (22)	0.983 (22)
	0.8	1.019 (16)	1.005 (15)	0.940 (14)	0.996 (15)	0.977 (15)	0.963 (15)	0.967 (15)
	1.0	1.024 (15)	1.007 (15)	0.952 (14)	1.000 (14)	0.986 (14)	0.967 (14)	0.968 (14)
	1.5	1.045 (15)	1.029 (14)	0.988 (14)	1.025 (14)	1.015 (14)	0.996 (14)	0.989 (14)
	2.0	1.047 (14)	1.031 (14)	1.000 (14)	1.030 (14)	1.021 (14)	1.006 (14)	0.994 (14)
	3.0	1.040 (13)	1.026 (13)	1.003 (13)	1.027 (13)	1.020 (13)	1.008 (13)	0.995 (13)
	4.0	1.029 (12)	1.017 (12)	0.998 (12)	1.018 (12)	1.013 (12)	1.002 (12)	0.991 (12)
	5.0	1.018 (11)	1.009 (11)	0.993 (11)	1.010 (11)	1.005 (11)	0.996 (11)	0.987 (11)
	10.0	0.999 (1)	0.999 (0)	0.999 (0)	0.999 (1)	0.999 (1)	0.999 (1)	0.999 (0)
10 MV WFF	0.5	0.979 (25)	0.975 (25)	0.896 (23)	0.979 (25)	0.938 (24)	0.947 (24)	0.969 (24)
	0.8	0.980 (14)	0.978 (14)	0.896 (13)	0.970 (14)	0.943 (14)	0.930 (14)	0.956 (14)
	1.0	0.990 (14)	0.986 (14)	0.915 (13)	0.977 (13)	0.957 (13)	0.938 (13)	0.961 (13)
	1.5	1.007 (13)	1.002 (13)	0.954 (12)	0.997 (13)	0.982 (12)	0.964 (12)	0.978 (12)
	2.0	1.010 (13)	1.003 (13)	0.971 (12)	1.000 (13)	0.990 (13)	0.975 (12)	0.982 (12)
	3.0	1.012 (12)	1.007 (12)	0.987 (12)	1.005 (12)	0.999 (12)	0.989 (12)	0.988 (12)
	4.0	1.013 (12)	1.007 (12)	0.994 (12)	1.007 (12)	1.002 (12)	0.995 (12)	0.993 (12)
	5.0	1.010 (11)	1.005 (11)	0.995 (11)	1.005 (11)	1.002 (11)	0.996 (11)	0.994 (11)
	10.0	1.000 (1)	1.000 (1)	1.000 (1)	1.000 (0)	1.000 (1)	1.000 (1)	1.000 (0)
10 MV FFF	0.5	0.982 (17)	0.977 (16)	0.919 (15)	0.984 (17)	0.953 (16)	0.955 (16)	0.977 (16)
	0.8	0.993 (12)	0.988 (12)	0.920 (11)	0.981 (12)	0.962 (12)	0.945 (12)	0.966 (12)
	1.0	1.011 (11)	1.005 (11)	0.944 (10)	0.997 (11)	0.982 (11)	0.962 (11)	0.980 (11)
	1.5	1.018 (10)	1.011 (10)	0.970 (9)	1.006 (10)	0.996 (9)	0.976 (9)	0.985 (9)
	2.0	1.016 (10)	1.008 (10)	0.979 (9)	1.005 (10)	0.999 (10)	0.982 (9)	0.986 (9)
	3.0	1.016 (9)	1.009 (9)	0.992 (9)	1.007 (9)	1.004 (9)	0.992 (9)	0.991 (9)
	4.0	1.017 (9)	1.010 (9)	0.998 (9)	1.010 (9)	1.008 (9)	0.999 (9)	0.996 (9)
	5.0	1.016 (9)	1.010 (9)	1.000 (9)	1.009 (9)	1.008 (9)	1.002 (9)	0.999 (9)
	10.0	0.999 (1)	0.999 (1)	0.999 (0)	0.999 (0)	0.999 (1)	0.999 (1)	0.999 (1)

a Field size is indicated as the nominal square field side length. The relationship between the nominal field size and the corresponding equivalent square small field sizes $S_{\mathrm{clin}}$ is provided in Table 2 for all energies and field sizes.

Tables X and XI provide $k_{\mathrm{vol}}$ for all detectors and energies on both linacs. Only fields with dimensions ≤1.5 cm were considered as no volume averaging effect was observed at larger field sizes. $k_{\mathrm{vol}}$ was found to be below 5% regardless of the detector, beam energy, field size, or linac. The largest $k_{\mathrm{vol}}$ were found for the PTW 60019 mD detector, which also has the largest active area perpendicular to the beam axis among all investigated detectors. In this case, $k_{\mathrm{vol}}$ range from 3.5% to 4.0% for the Elekta Versa HD, and from 3.9% to 4.4% for the Varian TrueBeam, for the smallest field of size of 0.5 cm.

**Table 10:** Volume averaging correction factors $k_{\mathrm{vol}}$ for six diodes and microdiamond detector calculated from 2D lateral field profiles obtained with EBT3 films for four smallest fields on the Elekta Versa HD

*E*	Field size a (cm)	IBA SFD diode	IBA Razor diode	PTW 60008 Diode P	PTW 60012 Diode E	PTW 60018 Diode SRS	SN EDGE Detector	PTW 60019 mD
6 WFF	0.5	1.003	1.003	1.012	1.012	1.012	1.007	1.040
	0.8	1.001	1.001	1.004	1.004	1.004	1.002	1.012
	1.0	1.001	1.001	1.002	1.002	1.002	1.001	1.007
	1.5	1.000	1.000	1.000	1.000	1.000	1.000	1.001
6 FFF	0.5	1.003	1.003	1.012	1.012	1.012	1.006	1.039
	0.8	1.001	1.001	1.003	1.003	1.003	1.002	1.011
	1.0	1.001	1.001	1.002	1.002	1.002	1.001	1.007
	1.5	1.000	1.000	1.000	1.000	1.000	1.000	1.001
10 WFF	0.5	1.003	1.003	1.010	1.010	1.010	1.006	1.035
	0.8	1.001	1.001	1.003	1.003	1.003	1.002	1.011
	1.0	1.000	1.000	1.002	1.002	1.002	1.001	1.006
	1.5	1.000	1.000	1.000	1.000	1.000	1.000	1.001
10 FFF	0.5	1.003	1.003	1.010	1.010	1.010	1.006	1.035
	0.8	1.001	1.001	1.003	1.003	1.003	1.001	1.009
	1.0	1.000	1.000	1.002	1.002	1.002	1.001	1.006
	1.5	1.000	1.000	1.000	1.000	1.000	1.000	1.002

a Field size is indicated as nominal square field side length. The relationship between the nominal field size and the corresponding equivalent square field sizes $S_{\mathrm{clin}}$ is provided in Table 2 for all energies and field sizes.

**Table 11:** Volume averaging correction factors $k_{\mathrm{vol}}$ for six diodes and microdiamond detector calculated from 2D lateral field profiles obtained with EBT3 films for four smallest fields on the Varian TrueBeam

E	Field size^a^ (cm)	IBA SFD diode	IBA Razor diode	PTW 60008 Diode P	PTW 60012 Diode E	PTW 60018 Diode SRS	SN EDGE Detector	PTW 60019 mD
6 WFF	0.5	1.003	1.003	1.013	1.013	1.013	1.007	1.044
	0.8	1.002	1.002	1.006	1.006	1.006	1.004	1.021
	1.0	1.000	1.000	1.002	1.002	1.002	1.001	1.005
	1.5	1.000	1.000	1.000	1.000	1.000	1.000	1.001
6 FFF	0.5	1.003	1.003	1.013	1.013	1.013	1.007	1.044
	0.8	1.001	1.001	1.002	1.002	1.002	1.001	1.007
	1.0	1.000	1.000	1.001	1.001	1.001	1.001	1.003
	1.5	1.000	1.000	1.000	1.000	1.000	1.000	1.001
10 WFF	0.5	1.003	1.003	1.011	1.011	1.011	1.006	1.039
	0.8	1.001	1.001	1.006	1.006	1.006	1.003	1.020
	1.0	1.001	1.001	1.002	1.002	1.002	1.001	1.007
	1.5	1.000	1.000	1.000	1.000	1.000	1.000	1.002
10 FFF	0.5	1.003	1.003	1.012	1.012	1.012	1.006	1.039
	0.8	1.001	1.001	1.005	1.005	1.005	1.003	1.016
	1.0	1.000	1.000	1.002	1.002	1.002	1.001	1.006
	1.5	1.000	1.000	1.000	1.000	1.000	1.000	1.002

a Field size is indicated as the nominal square field side length. The relationship between the nominal field size and the corresponding equivalent square small field sizes $S_{\mathrm{clin}}$ is provided in Table 2 for all energies and field sizes.

## 4 Discussion

### 4.A. Field output factors

In this study, field output factors for small fields were determined with two reference detectors, radiochromic EBT3 films, and Exradin W1 plastic scintillator, which are perturbation free except for volume averaging.^1^ The study was conducted on two different linacs, Elekta Versa HD and Varian TrueBeam, for four beam energies: 6 MV WFF, 6 MV FFF, 10 MV WFF, and 10 MV FFF. The measured data were corrected for volume averaging effect and fitted with the analytical function proposed by Sauer and Wilbert.^8^ Volume averaging correction factors $k_{\mathrm{vol}}$ are found to be almost negligible for EBT3 films (maximum values around 0.2%) while for W1 PSD they reach almost 1% for the smallest nominal field size of 0.5 cm (Tables 3 and 5). The data for W1 PSD indicate that volume averaging has to be considered in the determination of the field output factors. The present approach for calculating $k_{\mathrm{vol}}$ from two‐dimensional (2D) dose matrices obtained with EBT3 films is a viable option for correcting measured signals for volume averaging effect.

The analytical function from Eq. (14) yielded excellent fits to the measured data: uncertainties of the fit ranged from 1.0% to 1.4% (1 SD) for the smallest field size of 0.5 cm when measurements were performed on the Elekta Versa HD linac for the beam energies investigated. Results of measurements on the Varian TrueBeam exhibited higher uncertainties ranging from 1.6% to 2.5% (1 SD) for the smallest field size of 0.5 cm, which is possibly due to the higher inhomogeneity of EBT3 films in the lot that was used on the Varian TrueBeam linac.

As expected, a rapid decrease in field output factors was observed for field sizes below 2 cm, regardless of the beam energy or linac used. This is primarily due to the loss of lateral charged‐particle equilibrium and partial occlusion of the primary radiation source by different collimating devices used in the study, which is thoroughly described in the literature.^1^, ^37^, ^52^, ^53^

It can be seen from Table 4 that the field output factors for FFF beams of a given beam energy are always larger than the field output factors for the corresponding (same energy and linac) WFF beams for all investigated fields. The results of one‐tailed Student's *t*‐test given in Table 7 show that for 6 and 10 MV beams on the Elekta Versa HD and for 10 MV beams on the Varian TrueBeam, statistically significant differences (*P* < 0.05) are observed for field output factors between the FFF and the corresponding WFF beams for all investigated fields except for the 5 cm field for 6 MV beams on the Elekta Versa HD. For 6 MV beams, on the Varian TrueBeam, similar differences in the same level were found only for the smallest field of 0.5 cm. The present results suggest that in general, for a given linac, small field output factors will need to be determined individually for every combination of beam energy and filtration (WFF or FFF) and field size as the differences (Table 7) from each other are/can be statistically significant. Thus, the data presented in this study can potentially be used as a reference dataset for field output factors for the two linacs investigated.

The present novel method for determining field output factors requires significant expertise and high experimental skills in handling W1 PSD detectors and EBT3 films; it is time‐consuming and very demanding.

### 4.B. Detector‐specific output correction factors

Figures 4 and 5 show plots of the output correction factors vs equivalent square small field size $S_{\mathrm{clin}}$ for all six diodes and the microdiamond detector. An analysis of the graphs for $k(S_{\mathrm{clin}})$ shown in Figs. 4 and 5 show that the curves for all diodes follow a general pattern for all beam energies. As can be seen from the figures, for a given value of $S_{\mathrm{clin}}$, the curves for the output correction factors are at the top of the graphs for the IBA SFD diode, followed by the IBA Razor diode, PTW 60012 Diode E, PTW 60018 Diode SRS, SN EDGE detector, and PTW 60008 Diode P. The PTW 60019 microDiamond exhibits somewhat different behavior although its curves are very similar to the curves of the PTW 60018 Diode SRS, SN EDGE detector, and PTW 60008 Diode P.

At this point, we underline that the upcoming analysis and comparisons were strictly done for equivalent square field size $S_{\mathrm{clin}}$.

#### 4.B.1. Comparison with data given in TRS‐483

TRS‐483 has recently recommended values of output correction factors $k_{Q_{\mathrm{clin}},Q_{\mathrm{ref}}}^{f_{\mathrm{clin}},f_{\mathrm{ref}}}$ for many detectors for WFF and FFF beams for performing accurate relative dosimetry (i.e., measurements of field output factors) in high‐energy photon beams. Values of $k_{Q_{\mathrm{clin}},Q_{\mathrm{ref}}}^{f_{\mathrm{clin}},f_{\mathrm{ref}}}$ for different detectors are given as a function beam energy (i.e., 6 and 10 MV) as well as equivalent square small field size $S_{\mathrm{clin}}$. The data given in TRS‐483 for $k_{Q_{\mathrm{clin}},Q_{\mathrm{ref}}}^{f_{\mathrm{clin}},f_{\mathrm{ref}}}$ does not distinguish between linacs, filtration of beams (i.e., does not distinguish between WFF and FFF beams), and the types of collimation used, that is, output correction factors do not depend on whether the collimation is performed using MLC or SRS cones.

Comparisons were performed between the $k_{Q_{\mathrm{clin}},Q_{\mathrm{ref}}}^{f_{\mathrm{clin}},f_{\mathrm{ref}}}$ values obtained in this study and those recommended in TRS‐483 for six detectors for both the filtered (WFF) and unfiltered (FFF) 6 and 10 MV beams. TRS‐483 did not provide any data for the IBA Razor diode. Therefore, comparisons could not be performed for this detector.

For every beam energy, detector, and $S_{\mathrm{clin}}$ combination used in this study, the values of $k_{Q_{\mathrm{clin}},Q_{\mathrm{ref}}}^{f_{\mathrm{clin}},f_{\mathrm{ref}}}$ from TRS‐483 were obtained by linear interpolation of the corresponding data given in TRS‐483. A two‐tailed Student's *t*‐test was performed to evaluate the statistical significance of differences between the data for $k_{Q_{\mathrm{clin}},Q_{\mathrm{ref}}}^{f_{\mathrm{clin}},f_{\mathrm{ref}}}$ given in TRS‐483 and the corresponding data in this study. Unless stated otherwise, only statistically significant differences will be pointed out in the rest of the paper.

For the IBA SFD diode, statistically significant differences (*P* < 0.05) were found only for the smallest field of 0.5 cm on the Elekta linac for 6 MV FFF and 10 MV FFF beams.

For the PTW 60012 Diode P, statistically significant differences (*P* < 0.05) were found on both linacs for 10 MV WFF beams for small fields 1.5 and 2.0 cm. Comparison was not done for fields below 1.5 cm as the data for these fields are not provided in the TRS‐483.

For the PTW 60012 Diode E, statistically significant difference (*P* < 0.05) was found only for one beam, 6 MV WFF on the Elekta linac for the smallest field of 0.5 cm.

In the case of PTW 60018 Diode SRS, statistically significant differences (*P* < 0.05) were found on both linacs: on the Elekta linac for the smallest field of 0.5 cm for 6 MV WFF and FFF beams as well as for 0.8 cm field for 10 MV WFF beam while on the Varian linac only for 10 MV WFF beam for field size of 0.8 cm.

Comparison of $k_{Q_{\mathrm{clin}},Q_{\mathrm{ref}}}^{f_{\mathrm{clin}},f_{\mathrm{ref}}}$ values for the SN EDGE detector revealed statistically significant differences when 10 MV WFF beams are used on both linacs for small fields of 0.8, 1.0, 1.5, and 2.0 cm with *P*‐values of 0.033, 0.009, 0.013, and 0.024 for the Elekta linac and 0.033, 0.012, 0.026, and 0.034 for the Varian linac, respectively. Comparison was not performed for the 0.5 cm field, as the data for this field are not provided in the TRS‐483. A one‐tailed Student's *t*‐test was used in this case for the statistical evaluation as the $k_{Q_{\mathrm{clin}},Q_{\mathrm{ref}}}^{f_{\mathrm{clin}},f_{\mathrm{ref}}}$ values from this study were lower than the corresponding values from TRS‐483 for all field sizes.

Similar results were also observed when the values of $k_{Q_{\mathrm{clin}},Q_{\mathrm{ref}}}^{f_{\mathrm{clin}},f_{\mathrm{ref}}}$from TRS‐483 were compared with those obtained from this study for the PTW 60019 microDiamond detector. For this case, however, statistically significant differences (Table XII) were found for all beam energies on the Elekta Versa HD linac for fields ranging from 0.5 to 1.0 cm; additionally, for the 10 MV WFF and FFF beams, this behavior was observed for fields up to 2.0 cm. On the Varian linac, statistically significant differences (*P* < 0.05) were found only for the 10 MV WFF beam for small fields of 0.8 and 1.0 cm with *P*‐values of 0.033 and 0.022, respectively. Similar to the SN EDGE detector, the $k_{Q_{\mathrm{clin}},Q_{\mathrm{ref}}}^{f_{\mathrm{clin}},f_{\mathrm{ref}}}$ values of the PTW 60019 microDiamond detector from this study were found to be always lower than the corresponding values given in TRS‐483. A one‐tailed Student's *t*‐test was used to evaluate the statistical difference between the two sets of data.

**Table 12:** Statistical significance (*P*‐values) of differences between output correction factors $k_{Q_{\mathrm{clin}},Q_{\mathrm{ref}}}^{f_{\mathrm{clin}},f_{\mathrm{ref}}}$ for PTW 60019 mD detector given in TRS‐483 and the corresponding values from this study evaluated with a one‐tailed Student's *t*‐test. Data for 6 and 10 MV beams from TRS‐483 were compared with data from this study for both filtered (WFF) and unfiltered (FFF) 6 and 10 MV beams on the Elekta Versa HD linac

*P*‐values				
Field size^a^ (cm)	Elekta Versa HD			
	6 MV WFF	6 MV FFF	10 MV WFF	10 MV FFF
PTW 60019 mD				
0.5	0.001	0.003	0.001	0.001
0.8	0.027	0.025	0.001	0.004
1.0	0.014	0.014	0.002	0.010
1.5	0.070	0.059	0.016	0.027
2.0	0.118	0.042	0.023	0.031

a Field size is indicated as the nominal square field side length. The relationship between the nominal field size and the corresponding equivalent square field sizes $S_{\mathrm{clin}}$ is provided in Table 2 for all energies and field sizes.

#### 4.B.2. Influence of beam filtration and collimating system on output correction factors

As stated earlier, TRS‐483 provided tabulated data for output correction factors $k_{Q_{\mathrm{clin}},Q_{\mathrm{ref}}}^{f_{\mathrm{clin}},f_{\mathrm{ref}}}$ for several solid‐state detectors as a function of the equivalent square small field size $S_{\mathrm{clin}}$. These data do not distinguish between WFF and FFF beams for a given beam energy. Furthermore, the data also do not distinguish between the types of collimation that are used to create a small field, that is, whether the fields are collimated using MLC or SRS cones. To investigate the validity of these recommendations, an analysis was done to determine the dependence of the $k_{Q_{\mathrm{clin}},Q_{\mathrm{ref}}}^{f_{\mathrm{clin}},f_{\mathrm{ref}}}$ values for different detectors on beam collimation and beam filtration for a given beam energy.

First, for a given beam energy and a selected linac, all small field $k_{Q_{\mathrm{clin}},Q_{\mathrm{ref}}}^{f_{\mathrm{clin}},f_{\mathrm{ref}}}$ values for the investigated detectors for WFF and FFF beams were compared with each other to determine whether output correction factors differed significantly from each other or not. No significant differences were observed for measurements done with the IBA Razor, PTW 60012 E, and PTW 60019 mD detectors on both the Versa HD and TrueBeam linacs using both 6 MV WFF/6 MV FFF and 10 MV WFF/10 MV FFF beams. For the other four detectors, significant differences (*P* < 0.05) were found for the smallest field sizes when measurements were done on the Elekta Versa HD linac. Measurements with the IBA SFD diode showed significant differences using both 6 MV WFF/FFF and 10 MV WFF/FFF beams for field size 0.5 cm. In the case of PTW 60012 diode E, significant differences were found for 6 MV WFF/FFF beams for 0.5 cm field, while comparison of measurements performed with the PTW 60018 Diode SRS and SN Edge detector showed significant differences for 10 MV WFF/FFF beams for field sizes 0.8 and 0.5 cm, respectively. The diode PTW 60008 P showed significant dependence on beam filtration for 10 MV beams on the Varian linac; statistically significant differences (*P* < 0.05) were found for field sizes of 0.8 and 1.0 cm, and this was the only case where significant differences were found between WFF and FFF beams on the Varian linac.

Second, $k_{Q_{\mathrm{clin}},Q_{\mathrm{ref}}}^{f_{\mathrm{clin}},f_{\mathrm{ref}}}$ values for selected beam energy and filtration on one linac was compared to the corresponding values on the second linac, for example, $k_{Q_{\mathrm{clin}},Q_{\mathrm{ref}}}^{\;f_{\mathrm{clin}},\;f_{\mathrm{ref}}}$ values for 6 MV WFF beam on the Elekta linac were compared to the corresponding values for 6 MV WFF beam on the Varian linac, etc. Analysis of the results show that statistically significant differences (*P* < 0.05) were found for all detectors but the IBA Razor diode, predominantly for the smallest clinical field 0.5 cm for 6 MV WFF (PTW 60008 Diode P, PTW 60008 Diode E, PTW 60008 Diode SRS, SN EDGE, and PTW mD detectors) and 6 MV FFF beams (IBA SFD, PTW 60012 E, PTW 60008 Diode SRS, and PTW mD detectors).

For both the 10 MV WFF and 10 MV FFF beams, significant differences in $k_{Q_{\mathrm{clin}},Q_{\mathrm{ref}}}^{\;f_{\mathrm{clin}},\;f_{\mathrm{ref}}}$ values were found between the two linacs when the PTW 60019 mD detector was used for measurements in the smallest field of 0.5 cm. For this detector, observed differences for 0.5 cm field were 5.9% (*P* = 0.009), 5.5% (*P* = 0.019), 6.1% (*P* = 0.017), and 7.1% (*P* = 0.004) for 6 MV WFF, 6 MV FFF, 10 MV WFF, and 10 MV FFF beams, respectively. For all the other detectors, no statistically significant differences were observed for both the 10 MV WFF and 10 MV FFF beams.

It can then be concluded that different collimation system affects the output correction factors significantly for the 6 MV WFF and 6 MV FFF beams for the smallest investigated field size of 0.5 cm for all solid‐state detectors included in this study, with the only exception of IBA Razor diode for which no differences were seen. The PTW 60019 mD detector showed differences in output correction factors for the smallest field also for the 10 MV WFF and FFF beams. The other detectors did not show any beam collimation dependence for the 10 MV WFF and 10 MV FFF beams. These results show that for a given beam energy, the $k_{Q_{\mathrm{clin}},Q_{\mathrm{ref}}}^{f_{\mathrm{clin}},f_{\mathrm{ref}}}$ values obtained from different linacs (i.e., different collimation system) using different detectors can be different for $S_{\mathrm{clin}}<0.8\;\text{cm}$.

### 4.C. PTW 60019 mD detector

Properties of the PTW 60019 mD detector (mD) have been studied extensively, in particular, the determination of field output correction factors in small fields for combinations of various types of linacs, beam energies, and collimation systems. While the reported data for field output correction factors are reasonably consistent for field sizes of about 1 cm or larger, they diverge for field sizes below 1 cm.^54^ Remarkably, published data show specific pattern for the smallest fields around 0.5 cm. MC studies and hybrid studies (partly MC, partly experimental) report $k_{Q_{\mathrm{clin}},Q_{\mathrm{ref}}}^{f_{\mathrm{clin}},f_{\mathrm{ref}}}$ values which are close to unity or slightly higher,^25^, ^32^, ^34^, ^54^, ^55^, ^56^, ^57^ indicating that an under‐response (increase of $k_{Q_{\mathrm{clin}},Q_{\mathrm{ref}}}^{f_{\mathrm{clin}},f_{\mathrm{ref}}}$ values compared to the next larger field size) of the mD detector was observed for the smallest field size. On the contrary, in several experimental studies, authors have found a rather continuous increase of over‐response of the mD detector down to the smallest field sizes, yielding $k_{Q_{\mathrm{clin}},Q_{\mathrm{ref}}}^{f_{\mathrm{clin}},f_{\mathrm{ref}}}$ values, which are always few percent below unity.^9^, ^15^, ^21^, ^23^

Andreo et al. reported two different results within their MC study.^32^ First, they calculated field output correction factors for the mD detector following the manufacturer's blueprints of its design and components. In this part of the study, they found a similar response of the mD detector for the smallest field sizes as it was reported in several other MC studies.^25^, ^32^, ^34^, ^54^, ^55^, ^56^, ^57^ However, they found that the dimensions of the mD detector did not match those stated by the manufacturer, which brought them to repeat the calculations based on the new data for the active volume of the mD detector. Results from that part of the study were in close agreement with the experimental data.

In our study, $k_{Q_{\mathrm{clin}},Q_{\mathrm{ref}}}^{f_{\mathrm{clin}},f_{\mathrm{ref}}}$ values for the mD detector were below unity for all investigated field sizes, regardless of the beam energy, filtration, or linac used. Lowest $k_{Q_{\mathrm{clin}},Q_{\mathrm{ref}}}^{f_{\mathrm{clin}},f_{\mathrm{ref}}}$ values (largest corrections) were found for the Elekta Versa HD linac for 0.5 cm field size — 0.924 and 0.906 for the 6 and 10 MV beams, respectively, which represent an average of the dataset for the WFF and FFF beams. For the 6 MV beam, our values are around 3–4% lower than the corresponding values from TRS‐483 and values from previously mentioned experimental studies,^9^, ^15^, ^21^, ^23^ while for the 10 MV beam, the present data for $k_{Q_{\mathrm{clin}},Q_{\mathrm{ref}}}^{f_{\mathrm{clin}},f_{\mathrm{ref}}}$ are lower than corresponding data reported in TRS‐483 by close to 6% for 0.5 cm field size. These differences are outside the uncertainties reported in both studies. For the Varian TrueBeam, corresponding $k_{Q_{\mathrm{clin}},Q_{\mathrm{ref}}}^{f_{\mathrm{clin}},f_{\mathrm{ref}}}$ values are 0.983 and 0.973 for the 6 and 10 MV beams, respectively (Table 9), which is around 1–2% higher compared to data from TRS‐483, however, within the reported uncertainties. On the Elekta linac, we noticed continuous over‐response ($k_{Q_{\mathrm{clin}},Q_{\mathrm{ref}}}^{f_{\mathrm{clin}},f_{\mathrm{ref}}}<1$) of the mD detector down to the smallest field size, while it was not the case for the Varian linac, where $k_{Q_{\mathrm{clin}},Q_{\mathrm{ref}}}^{f_{\mathrm{clin}},f_{\mathrm{ref}}}$ for 0.5 cm field size were always higher than those for the 0.8 cm field size for all beam energies; we attributed this to the type of linac and different collimating systems used. It is important to note that for the mD detector, published values for $k_{Q_{\mathrm{clin}},Q_{\mathrm{ref}}}^{f_{\mathrm{clin}},f_{\mathrm{ref}}}$ in TRS‐483 are exactly the same for the 6 and 10 MV beams, which suggests, that there is no distinction in the field output correction factors for the mD detector regardless of the beam energy, filtration, collimation system, and linac, an observation, which was not confirmed in our work.

In this study, differences of up to 6% for 6 MV and close to 7% for 10 MV were observed for field output correction factors for 0.5 cm field size when measurements were made using the mD detector in the Varian and Elekta linacs. This suggests that for field sizes below 1 cm, field output correction factors for the mD detector depends on the combination of linac type, beam energy, and beam collimation system used. It is worth noting that similar but less pronounced differences in field output correction factors were also observed for the smallest field size for two different collimation systems in the experimental study by Underwood et al.^21^

To summarize, the results of our experimental study show that $k_{Q_{\mathrm{clin}},Q_{\mathrm{ref}}}^{f_{\mathrm{clin}},f_{\mathrm{ref}}}$ values for the mD detector are below unity for field sizes below 1 cm, regardless of the linac type, beam collimation system, and beam energy or filtration used; this confirms observed over‐response (regardless of the field size) from several experimental studies,^9^, ^15^, ^21^, ^23^ as well as the MC study by Andreo et al.^32^ Moreover, the present results also suggest that the mD detector cannot be considered as an almost correction‐less detector for small field dosimetry; additionally, the field output correction factors for this detector depend on the type of linac, beam energy, and collimations used.

## 5 Summary

This paper presents results of field output factors for small photon fields determined by two reference detectors, radiochromic films EBT3 and Exradin W1 plastic scintillator, which are perturbation free except for volume averaging. Results are presented as analytical functions as well as discrete values for nine fields, ranging from 0.5 × 0.5 cm^2^ to 10 × 10 cm^2^. Measurements were made on the Varian TrueBeam and Elekta Versa HD linacs using the 6 MV WFF, 6 MV FFF, 10 MV WFF, and 10 MV FFF beams. Only volume averaging correction factors $k_{\mathrm{vol}}$ were applied to the measured datasets; these were calculated from 2D dose matrices obtained from EBT3 films for each small field and beam energy individually, which were fitted to bivariate Gaussian function. This is a novel approach for the determination of field output factors in small static fields in megavoltage photon beams. Field output factor data presented in this study can be used as reference datasets on linacs with same collimation of the fields as was used in this study.

Additionally, based on calculated field output factors, detector‐specific output correction factors $k_{Q_{\mathrm{clin}},Q_{\mathrm{ref}}}^{f_{\mathrm{clin}},f_{\mathrm{ref}}}$were determined for six diodes and a microdiamond detector, which are widely used for performing relative dosimetry in the clinics.

Large set of field output factor and output correction factor data for seven detectors and four beam energies were determined/measured on two linacs by a single group; this is considered to be a valuable supplement to the literature and to the TRS‐483 dataset. Data are presented in graphical form using an analytical function from TRS‐483 as well as in the form of discrete values.

Comparison between the output correction factors reported in the present work and those published in TRS‐483 show statistically significant difference (*P* < 0.05) when a SN EDGE detector is used for measurements made with 10 MV WFF beams at both the Varian TrueBeam and Elekta Versa HD linacs for field sizes <2 cm. Statistically significant differences (*P* < 0.05) were also found when a PTW 60019 mD detector was used with the 6 MV WFF, 6 MV FFF and 10 MV FFF beams on the Elekta Versa HD linac. These findings are different from the recommendations given in TRS‐483.

For most combinations of field size, beam energy, and beam collimation, no significant differences were found between TRS‐483 dataset and the present results for output correction factors for WFF and FFF beams; a few exceptions were found for the smallest field sizes.

Results of this study also show that different collimation systems significantly influence the output correction factors for the smallest field size of 0.5 × 0.5 cm^2^ for the 6 MV WFF and 6 MV FFF beams regardless of the detector used. For the PTW 60019 mD detector, this effect was observed also for the 10 MV WFF and 10 MV FFF beams. The only exception was IBA Razor diode for which dependence of output correction factors on beam collimating system was not observed. These results show that for a given beam energy, the $k_{Q_{\mathrm{clin}},Q_{\mathrm{ref}}}^{f_{\mathrm{clin}},f_{\mathrm{ref}}}$ values obtained from different linacs (i.e., different collimation system) using different detectors can be different for $S_{\mathrm{clin}}<0.8\;\text{cm}$.
